# Anxiety enhances pain in a model of osteoarthritis and is associated with altered endogenous opioid function and reduced opioid analgesia

**DOI:** 10.1097/PR9.0000000000000956

**Published:** 2022-02-03

**Authors:** Amanda Lillywhite, Stephen G. Woodhams, Sara V. Gonçalves, David J.G. Watson, Li Li, James J. Burston, Peter R.W. Gowler, Meritxell Canals, David A. Walsh, Gareth J. Hathway, Victoria Chapman

**Affiliations:** a Pain Centre Versus Arthritis, University of Nottingham, Medical School, Queen's Medical Centre, Nottingham, United Kingdom; b School of Life Sciences, Medical School, Queen's Medical Centre, Nottingham, United Kingdom; c Centre of Membrane Proteins and Receptors, Universities of Birmingham and Nottingham, Midlands, United Kingdom; d School of Medicine, University of Nottingham, Nottingham, United Kingdom; e NIHR Nottingham Biomedical Research Centre, University of Nottingham, Nottingham, United Kingdom

**Keywords:** Anxiety, Arthritis, Pain, Opioids, Animal models

## Abstract

**Introduction:**

Negative affect, including anxiety and depression, is prevalent in chronic pain states such as osteoarthritis (OA) and associated with greater use of opioid analgesics, potentially contributing to present and future opioid crises.

**Objectives:**

We tested the hypothesis that the interaction between anxiety, chronic pain, and opioid use results from altered endogenous opioid function.

**Methods:**

A genetic model of negative affect, the Wistar–Kyoto (WKY) rat, was combined with intra-articular injection of monosodium iodoacetate (MIA; 1 mg) to mimic clinical presentation. Effects of systemic morphine (0.5–3.5 mg·kg^−1^) on pain behaviour and spinal nociceptive neuronal activity were compared in WKY and normo-anxiety Wistar rats 3 weeks after MIA injection. Endogenous opioid function was probed by the blockade of opioid receptors (0.1–1 mg·kg^−1^ systemic naloxone), quantification of plasma β-endorphin, and expression and phosphorylation of spinal mu-opioid receptor (MOR).

**Results:**

Monosodium iodoacetate–treated WKY rats had enhanced OA-like pain, blunted morphine-induced analgesia, and greater mechanical hypersensitivity following systemic naloxone, compared with Wistar rats, and elevated plasma β-endorphin levels compared with saline-treated WKY controls. Increased MOR phosphorylation at the master site (serine residue 375) in the spinal cord dorsal horn of WKY rats with OA-like pain (*P* = 0.0312) indicated greater MOR desensitization.

**Conclusions:**

Reduced clinical analgesic efficacy of morphine was recapitulated in a model of high anxiety and OA-like pain, in which endogenous opioid tone was altered, and MOR function attenuated, in the absence of previous exogenous opioid ligand exposure. These findings shed new light on the mechanisms underlying the increased opioid analgesic use in high anxiety patients with chronic pain.

## Abstract 2

Supplemental Digital Content is Available in the Text.

Comorbid anxiety and osteoarthritis pain is associated with increased opioid intake. Our translational model identifies reduced opioid analgesia and endogenous opioid dysfunction as potential mechanisms.

## 1. Introduction

Chronic pain is a major clinical problem with limited treatment options. Since the 1990s, clinical use of opioids shifted from managing acute pain and pain in terminally ill patients, to wide-spread prescribing^19^ for long-term pain conditions, despite limited usefulness in most people.^6,17^ Opioid drugs, such as morphine, predominantly produce their effects through µ-opioid receptors (MOR) at key sites in the spinal cord and brain.^39^ The effects of chronic pain states on endogenous opioid function include increased release of endogenous opioid peptides, such as β-endorphin, alterations in MOR function, and lower analgesic responsiveness to morphine.^12,45^

Osteoarthritis (OA) is the fastest growing cause of chronic pain worldwide,^25,57^ underpinned by both nociceptive and neuropathic mechanisms^26,44^ and central sensitization in ∼20% of people with OA pain.^3,51^ Despite high rates of opioid-prescribing for OA pain,^10,53^ they have no superior effect over nonopioid treatments over 12 months,^33^ and long-term opioid use is associated with increased risk of adverse events.^10^

Negative affect is associated with exacerbated chronic pain^20^ and is common in people with OA,^5,8^ and complex relationships between endogenous opioid function and depressive symptoms and trait anxiety have been reported.^14^ Negative affect is associated with the greater use of opioid analgesics in people with OA.^8,41,56^ We hypothesised that the interaction between high anxiety, chronic pain, and increased opioid use in people may result from altered endogenous opioid function, which could be tested in a clinically relevant animal model.

Rodent models and molecular and pharmacological studies of opioid receptor function have advanced knowledge of pain mechanisms and opioid-induced analgesia.^42,43^ Inbred Wistar Kyoto (WKY) rats are an experimental model of anxiety-like behaviour.^38^ We reported increased magnitude and spread of pain behaviour in a model of OA pain in WKY rats,^15^ replicating the clinical association between anxiety and exacerbated OA pain. In this article, we measured opiate-mediated analgesia in a clinically relevant model of OA-like pain in WKY rats and normo-anxiety Wistar rats. Effects of morphine on spinal cord neuronal excitability and antagonist-mediated blockade of opioid receptors were used to probe endogenous opioid system function in this model. Measurements of plasma levels of β-endorphin and spinal cord levels of MOR protein and MOR receptor phosphorylation at serine residue 375 (P-ser-375), which is required for opioid-mediated desensitization,^50^ provided important new mechanistic insights.

## 2. Materials & methods

### 2.1. Experimental animals

Studies were conducted in accordance with UK Home Office Animals (Scientific Procedures) Act (1986) and ARRIVE guidelines.^31^ 211 male rats were used: Wistar n = 101 (Charles River, Margate, United Kingdom) and Wistar Kyoto n = 110 (WKY; Envigo, Bicester, United Kingdom). The relationship between anxiety and clinical prescription opioid use is stronger in males than females^47^; therefore, this study was restricted to male rats, although we recognise this as a limitation. Wistar rats are the most genetically similar control strain to WKY. Rats were group housed by strain, 4 per cage in a specific pathogen-free environment with a 12-hour light or dark cycle and ad libitum access to food and water. Treatments were assigned randomly, with experimenters blinded throughout the study. 17 rats were excluded from the study (8.1%; see Supplemental Table 1 for further details, available at http://links.lww.com/PR9/A130).

### 2.2. Induction of the monosodium iodoacetate model of osteoarthritis pain

Rats received a single intra-articular injection, randomly assigned to either 1 mg/50 µL MIA in 0.9% saline (Wistar n = 54 and WKY n = 51) or 50 µL 0.9% saline (Wistar n = 47 and WKY n = 47), through the infrapatellar ligament into the left knee, under isoflurane anaesthesia (3% in 1L.min^−1^ O2^48^). 12 naïve WKY animals were also used in this study (see 2.3 below). Health and welfare checks were performed immediately after anaesthetic recovery, daily for 3 days, and weekly thereafter. Pain behaviour was assessed twice weekly from D3 to 21.

### 2.3. Behavioural testing

**Figure 1.**
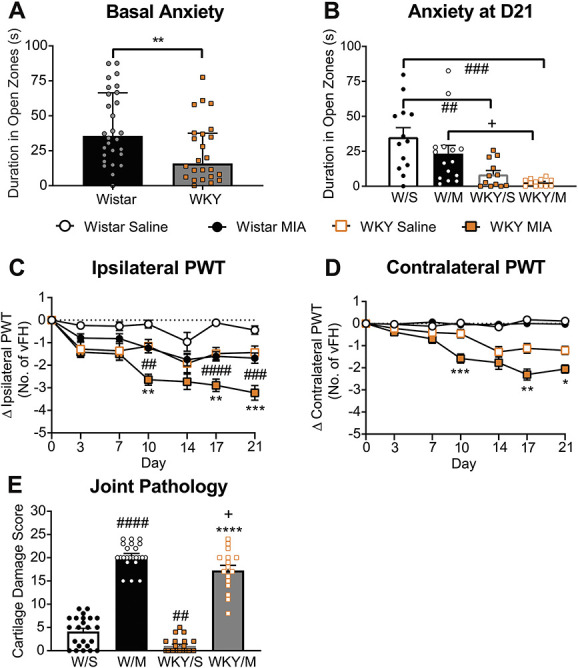
Anxiety-like phenotype and exacerbated OA-like pain in the WKY-MIA model. At baseline, WKY rats spent significantly less time in the open arms of the elevated plus maze (A). Bars indicate median values, and error bars represent IQR. **P < 0.01 vs Wistar rats, one-tailed Mann–Whitney *U* test (Supplemental Table 3, available at http://links.lww.com/PR9/A130). Three weeks after model induction, there was no significant effect of treatment on duration spent in the open arms of the elevated plus maze (B). MIA-treated and saline-treated WKY rats had a significant anxiety-like phenotype, compared with their respective Wistar groups. Data represent total duration in the open arms for each 10 minutes trial. Individual data points are shown, and bars represent the mean and SEM. ##*P* = 0.0032, ###*P* < 0.003 vs Wistar/saline, + *P* = 0.0246 vs Wistar/MIA, 2-way ANOVA with the Tukey multiple comparison *post hoc* test (Supplemental Table 6, available at http://links.lww.com/PR9/A130). MIA-treated rats had lowered ipsilateral PWT in WKY and Wistar rats (C). Contralateral PWTs were unaltered in MIA-injected or saline-injected Wistar rats but significantly lowered in MIA-treated WKY rats (D). Data are mean ± SEM change in vFH compared with baseline, *P < 0.05, **P < 0.01, ***P < 0.001 WKY/MIA vs WKY/saline, ##*P* < 0.01, ###*P* < 0.001, ####*P* < 0.0001 Wistar/MIA vs Wistar/saline, mixed-effects model analysis with the Tukey multiple comparison *post hoc* test (Supplemental Table 7, available at http://links.lww.com/PR9/A130). Macroscopic assessment of cartilage damage in ipsilateral knee joints in MIA-injected WKY and Wistar rats. Little or no cartilage damage was observed in saline-injected rats (E). Data represent the summed scores for each of the 5 individual joint compartments (0–5, max score 25). Individual data points are shown, and bars represent the mean and SEM. ##*P* = 0.0054, ####*P* < 0.0001 vs Wistar/saline, ****P < 0.0001 vs WKY/saline, + *P* = 0.0123 vs Wistar/MIA, 2-way ANOVA with the Tukey multiple comparison *post hoc* test (Supplemental Table 6, available at http://links.lww.com/PR9/A130). ANOVA, analysis of variance; MIA, monosodium iodoacetate; OA, osteoarthritis; PWTs, paw withdrawal thresholds; vFH, von Frey hair; WKY, Wistar Kyoto.

Pain behaviour was assessed by weight-bearing (WB) asymmetry using an incapacitance tester^11^ (Linton Instrumentation, Diss, United Kingdom). Weight-bearing asymmetry was calculated as [ipsilateral g/(ipsilateral g + contralateral g)]. Mechanical hind paw withdrawal thresholds (PWTs) were determined by von Frey hair (vFH) monofilaments using the up-down method.^16^ As the intervals between successive vFH are logarithmic,^40^ PWTs are reported as log vFH values to avoid biasing statistical comparisons. To enable pooling of experimental studies (see Supplemental Table 2, available at http://links.lww.com/PR9/A130) and preserve the interval nature of the vFH scale, PWTs were converted to change in the number of vFHs from baseline for Figure 1.

To measure anxiety-like behaviour, the total time spent in closed vs open arms in the elevated plus maze (EPM^58^) over a period of 10 minutes was quantified using EthoVision software (Noldus Information Technology, Netherlands).

To measure the locomotor activity, the number of beam breaks over 30 minutes in activity boxes (Photobeam Activity System; San Diego Instruments) was assessed at baseline and 19 to 21 days after model induction.^46^ To rule out effects of morphine on motor activity, the locomotor activity was assessed 60 to 90 minutes after the last of 3 consecutive doses of morphine (0.5, 2, and 3.5 mg·kg·mL^−1^, s.c.; n = 6) or saline (50 µL; n = 6) in a separate cohort of WKY rats.

### 2.4. Pharmacological interventions

#### 2.4.1. Systemic morphine or naloxone or CTAP behavioural study

To assess differences in opioid sensitivity or alterations in endogenous opioidergic tone, behavioural nociceptive responses after consecutive subcutaneous (s.c.) doses of morphine, the nonselective opioid receptor antagonist naloxone, and the MOR-selective antagonist D-Phe-Cys-Tyr-D-Trp-Arg-Thr-Pen-Thr-NH2 (CTAP^1^) or vehicle (0.9% saline) were determined in separate groups of Wistar and WKY rats 21 days after model induction. Pain behaviour was assessed in MIA-treated or saline-treated rats predrug and then after the following treatments: study 1—morphine (0.5, 2, and 3.5 mg·kg·mL^−1^, s.c.; Wistar/MIA n = 10, WKY/MIA n = 10) or vehicle (Wistar/saline n = 8 and WKY/saline n = 8); study 2— naloxone (0.1, 0.3, and 1 mg·kg·mL^−1^, s.c.; Wistar/saline n = 12, Wistar/MIA n = 12, WKY/saline n = 10, and WKY/MIA n = 11); study 3—CTAP (0.1, 0.3, and 1 mg·kg^−1^·mL^−1^,i.p.; Wistar/MIA n = 10 and WKY/MIA n = 10). Drug treatments were given at 60 minute intervals, and pain behaviour assessed at 15, 30, and 60 minutes after each dose (study 1 and 2), or 30 minutes only after each dose (study 3) (based on previous published literature^9,37^). % morphine analgesia was calculated for weight-bearing, with 100% analgesia equalling total normalisation of weight-bearing asymmetry. For effects of drug treatments on PWT, data are reported as logPWT values.

#### 2.4.2. In vivo spinal electrophysiology

Responses of deep dorsal horn wide dynamic range (WDR) neurons to hind paw stimulation were recorded 21 days after model induction (Wistar/saline n = 10, Wistar/MIA n = 15, WKY/saline n *=* 12, and WKY/MIA n = 12) as previously described^55^ (see Supplementary Methods, available at http://links.lww.com/PR9/A130). WDR neuronal responses to mechanical hind paw stimulation with 8, 10, 15, and 26 g vFH (10 seconds application and 10 seconds interstimulus interval) were recorded at 10 minute intervals to establish baseline responses and then every 10 minutes after consecutive doses of morphine sulfate (0.5, 2, and 3.5 mg·kg^−1^, s.c., 60 minute intervals).

Responses of WDRs were binned according to poststimulus latency for major primary afferent fibres (Aβ 0–20 ms, Aδ 20–90 ms, C 90–300 ms, and post-stimulus discharge 300–800 ms). Degree of wind-up was determined as the total number of C fibre and post-stimulus discharge spikes after a train of 16 stimuli (3x C-fibre threshold, 0.5 Hz). For mechanical stimuli, average firing rates (Hz) in response to 10 seconds stimulation with each vFH were recorded. The mean maximal inhibition (MMI) was calculated as maximal % change vs baseline for each dose of morphine and plotted by strain and treatment for each dose. For each vFH, the area under the curve (AUC) was calculated from MMI values for each dose for each individual animal and animals grouped by strain and treatment and compared in Prism 8.

### 2.5. Assessment of opioid function in ex vivo tissues

#### 2.5.1. β-endorphin ELISA

Levels of the endogenous opioid peptide β-endorphin were measured in plasma from male Wistar (n *=* 18) and WKY (n *=* 18) rats from study 3 using a commercially available enzyme-linked immunosorbent assay (ELISA) kit (Phoenix Pharmaceuticals, Burlingame, CA) according to the manufacturer's instructions (see Supplemental Methods, available at http://links.lww.com/PR9/A130).

#### 2.5.2. Western blotting

Fresh spinal cord tissue was collected from WKY and Wistar rats 21 days after MIA or saline treatment (cohort 3, n = 4/strain) and probed for the expression of total MOR (rabbit anti–mu-opioid receptor, Neuromics, RA10104, 1:500), P-ser375 MOR (rabbit anti–mu-opioid receptor Ser375, BIOSS-Stratech, bs-3724R, 1:500), and β-actin (mouse anti–β-actin, Sigma, A5441, 1:5000) using Western blotting (see Supplemental Methods, available at http://links.lww.com/PR9/A130).

### 2.6. Assessment of joint pathology

Knee joints from each animal were fixed in 10% neutral buffered formalin (48 hours), disarticulated, and macroscopic cartilage damage scored by a blinded experimenter by established methods.^28^ 7% of rats were excluded due to joint pathology inconsistent with the treatment group (see Supplemental Table 1, available at http://links.lww.com/PR9/A130).

### 2.7. Statistical analyses

Power calculations based on data from a similar previous study in this model^15^ were used to determine appropriate group sizes (see Supplemental Methods, available at http://links.lww.com/PR9/A130). Data were analysed using Prism 8 (GraphPad, La Jolla, CA). Data distributions were assessed by Shapiro–Wilk normality testing, and treated as parametric or nonparametric, as appropriate. Further statistical details are provided in Supplemental Methods (available at http://links.lww.com/PR9/A130).

## 3. Results

### 3.1. Wistar Kyoto rats exhibit a basal anxiety-like phenotype and exacerbated pain behaviour in the monosodium iodoacetate model of osteoarthritis

Wistar Kyoto rats spent significantly less time in the open arms of the elevated plus maze (EPM) when compared with Wistar rats (Fig. 1A, Supplemental Table 3, available at http://links.lww.com/PR9/A130). These data support a basal anxiety-like phenotype in the WKY strain. There was a significant effect of strain (Fig. 1B, Supplemental Table 6**,** available at http://links.lww.com/PR9/A130, P < 0.0001), but a nonsignificant effect of treatment (*P* = 0.086) on the time spent in the open arms of the EPM, consistent with our previous work.^15^ Locomotor activity was comparable between strains (Supplemental Figure 1C, available at http://links.lww.com/PR9/A130).

WKY and Wistar rats exhibited comparable hind paw withdrawal thresholds (PWTs), and weight was borne equally on both hind paws before any intervention (Supplemental Table 2, available at http://links.lww.com/PR9/A130). Consistent with previous work,^15^ ipsilateral PWT were lowered in both strains of rats (Fig. 1C), from day 10 after intra-articular injection of MIA. A significantly greater reduction in ipsilateral PWTs was observed in WKY rats, compared with Wistar rats (Supplemental Tables 2 and 6, available at http://links.lww.com/PR9/A130), with a reduction in contralateral PWTs in WKY rats alone (Fig. 1D, Supplemental Table 7, available at http://links.lww.com/PR9/A130), consistent with the presence of central sensitization and an exacerbated OA-like pain phenotype in WKY rats.^15^ Rats treated with MIA exhibited weight-bearing asymmetry in both strains of rats (Supplemental Figure 1A, available at http://links.lww.com/PR9/A130); this was less pronounced in the WKY strain, presumably because of lowered contralateral pain PWTs confounding this measure in a similar manner to models of neuropathic pain.

### 3.2. Osteoarthritis-like joint pathology

There were significant increases in cartilage damage in the MIA model in both WKY and Wistar rats (Fig. 1E, Supplemental Table 6, available at http://links.lww.com/PR9/A130). It is noteworthy that, despite the exacerbated pain phenotype, there was significantly less cartilage damage observed in WKY rats compared with Wistar rats.

### 3.3. Reduced effects of systemic morphine in the Wistar Kyoto-monosodium iodoacetate model of high anxiety and osteoarthritis-like pain behaviour

**Figure 2.**
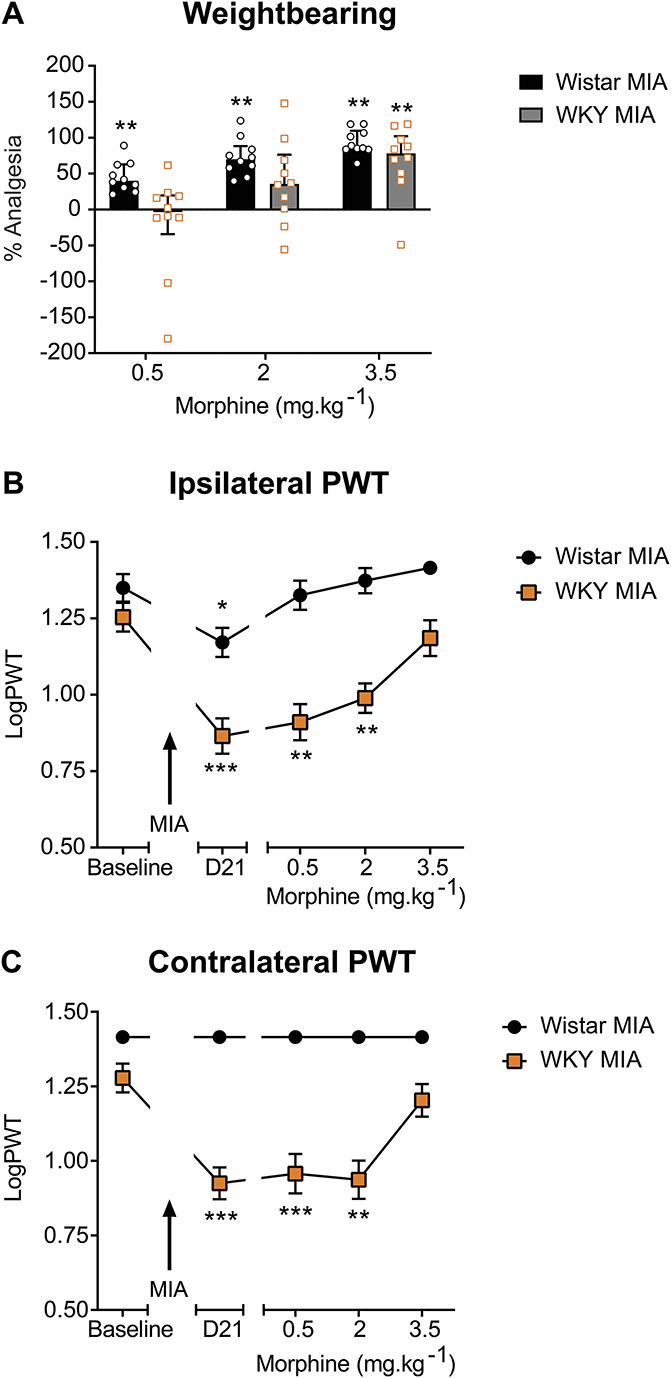
Reduced behavioural response to systemic morphine in the WKY-MIA model of high anxiety and OA-like pain. Systemic administration of morphine produced dose-related inhibition of weight-bearing asymmetry in the MIA model in Wistar rats (A), inhibitory effects of low dose morphine were significantly attenuated in WKY rats. Data represent % analgesia to 3 consecutive doses of systemic morphine, with abolition of weight-bearing asymmetry representing 100% analgesia **P = 0.01, Wilcoxon signed-rank test with a hypothetical value of 0 (Supplemental Table 5, available at http://links.lww.com/PR9/A130). The lowest dose of morphine restored MIA-induced decreases in ipsilateral PWTs in Wistar, but not WKY, rats (B). Morphine did not significantly alter contralateral PWTs in MIA-injected Wistar rats; the highest dose of morphine reversed lowered contralateral PWTs in MIA-injected WKY rats (C). Data represent mean ± SEM logPWT values. *P < 0.05, **P < 0.01, ***P < 0.001 vs baseline, repeated measures 2-way ANOVA with Dunnett multiple comparison post hoc testing (Supplemental Table 6, available at http://links.lww.com/PR9/A130). ANOVA, analysis of variance; MIA, monosodium iodoacetate; OA, osteoarthritis; PWTs, paw withdrawal thresholds; vFH, von Frey hair; WKY, Wistar Kyoto.

Systemic administration of morphine (0.5, 2, and 3.5 mg·kg^−1^ s.c.) produced a significant, dose-related reduction in weight-bearing asymmetry in Wistar rats (Fig. 2A, Supplemental Table 5, available at http://links.lww.com/PR9/A130). By contrast, only the highest dose of morphine significantly inhibited weight-bearing asymmetry in MIA-treated WKY rats (Fig. 2A). Similarly, morphine had a significantly blunted inhibitory effect on lowered ipsilateral PWTs in MIA-treated WKY rats, compared with the Wistar strain (Fig. 2B, Supplemental Table 6, available at http://links.lww.com/PR9/A130). All 3 doses of morphine significantly restored PWT deficits in MIA-treated Wistar rats, whereas only the highest dose of morphine produced significant reversal of ipsilateral PWTs in WKY rats (Fig. 2B, Supplemental Table 6, available at http://links.lww.com/PR9/A130). The highest dose of morphine also significantly reversed the lowered contralateral PWT evident in MIA-treated WKY rats (Fig. 2C, Supplemental Table 6, available at http://links.lww.com/PR9/A130). The assessment of locomotor activity after the dosing paradigm confirmed that the blunted effects of morphine in WKY rats were not due to morphine-induced suppression of locomotor activity in WKY rats (Supplemental Figure 2A&B, available at http://links.lww.com/PR9/A130).

### 3.4. Evidence for altered systemic endogenous opioid signalling in Wistar Kyoto rats

**Figure 3.**
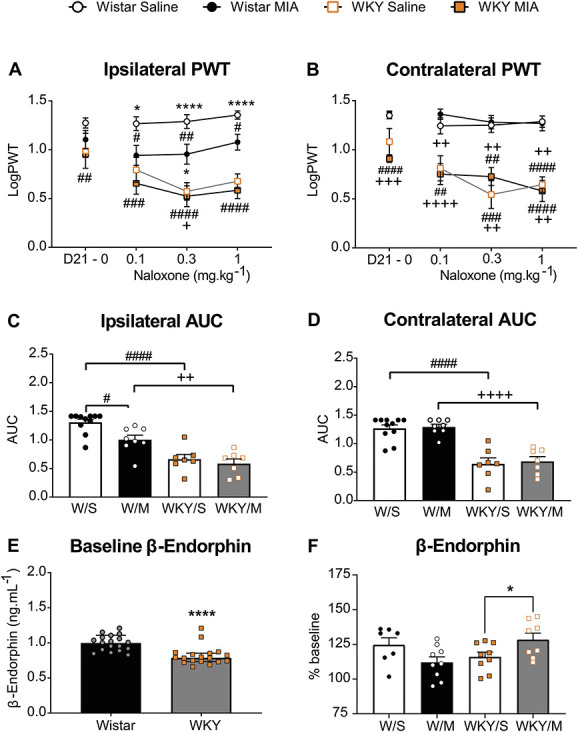
Altered endogenous opioid tone in rats with an anxiety-like phenotype. Systemic administration of the MOR antagonist naloxone (0.1–1 mg·kg^−1^, s.c.) significantly lowered ipsilateral PWTs in MIA-injected, but not saline-injected, Wistar rats (A). In both saline-treated and MIA-treated WKY rats, naloxone produced a significant, dose-dependent, and bilateral lowering of PWTs (A and B). Data represent mean ± SEM for baseline PWTs and MMI values for each consecutive dose of naloxone. #*P* < 0.05, ##*P* < 0.01, ###*P* < 0.001, ####*P* < 0.0001 vs Wistar/saline; *P < 0.05, ****P < 0.0001 vs WKY/saline; + *P* < 0.05, ++ *P* < 0.01, +++ *P* < 0.001, ++++ *P* < 0.0001 vs Wistar/MIA, 2-way ANOVA with Tukey multiple comparison *post hoc* testing (Supplemental Table 6, available at http://links.lww.com/PR9/A130). LogPWTs were plotted for each dose and the AUC calculated for each individual animal via Prism. These data revealed a significantly greater effect of naloxone on ipsilateral (C) and contralateral (D) PWTs in WKY rats when compared with Wistar rats. Individual data points; bars represent mean and SEMs. #*P* < 0.0167, ####*P* < 0.0001 vs Wistar/saline; ++ *P* = 0.026, ++++ *P* < 0.0001 vs Wistar/MIA, 2-way ANOVA with Tukey multiple comparison *post hoc* testing (Supplemental Table 6, available at http://links.lww.com/PR9/A130). Baseline plasma levels of β-endorphin were measured by ELISA in samples collected from naïve Wistar (n = 18) and WKY (n *=* 18) rats (E). Bars represent the mean value, error bars indicate SEM. ****P =<0.0001, unpaired *t* test (Supplemental Table 3, available at http://links.lww.com/PR9/A130). At 21 days after injection of MIA, plasma levels of β-endorphin were higher in WKY rats (n = 9) but not Wistar rats (*n* = 8–9). Bars represent the mean value, and error bars indicate SEM *P = 0.044, unpaired *t* test (Supplemental Table 4, available at http://links.lww.com/PR9/A130). ANOVA, analysis of variance; MIA, monosodium iodoacetate; MOR, µ-opioid receptors; OA, osteoarthritis; PWTs, paw withdrawal thresholds; vFH, von Frey hair; WKY, Wistar Kyoto.

We hypothesised that the reduced inhibitory effects of morphine on pain behaviour in WKY rats may arise as a result of changes in opioid receptor function or circulating levels of endogenous opioids. The effects on pain behaviour of blocking the µ-opioid receptor with the antagonist naloxone were assessed in the MIA model in both strains of rats (Fig. 3). Naloxone (0.1–1 mg·kg^−1^ s.c.) did not alter PWTs in Wistar rats in the absence of the model of OA-like pain (intra-articular injection of saline), suggesting no overt basal endogenous opioidergic tone in these rats. However, all 3 doses of naloxone (0.1–1 mg·kg^−1^) significantly lowered ipsilateral PWTs in MIA-treated Wistar rats, suggesting the presence of endogenous opioid tone after the induction of the model of OA pain in this strain of rats. In WKY rats, naloxone (0.1–1 mg·kg^−1^) produced a significant bilateral lowering of PWTs (Fig. 3A–D, Supplemental Table 6, available at http://links.lww.com/PR9/A130), which was similar in both saline-treated and MIA-treated WKY rats. These effects were mirrored in animals treated with the selective MOR antagonist, CTAP (Supplemental Figures 3A&B, available at http://links.lww.com/PR9/A130), supporting an effect mediated by µ-opioid receptors. These data suggest the presence of altered endogenous opioid peptides, or increased constitutive activity of MOR, in WKY rats in the absence of the OA pain model, which is not further increased in the presence of the model.

Plasma levels of β-endorphin were assayed in tail vein blood from both strains of rats collected under brief isoflurane anaesthesia. At baseline, plasma β-endorphin was significantly lower in WKY rats, compared with Wistar rats (Fig. 3E, Supplemental Table 3, available at http://links.lww.com/PR9/A130). At 21 days after intra-articular injection of saline, plasma levels of β endorphin were higher in both Wistar and WKY rats compared with baseline (Fig. 3F). At 21 days after intra-articular injection of MIA in Wistar rats, plasma levels of β-endorphin were comparable with levels in saline-treated Wistar rats at this timepoint (Fig. 3F). At 21 days after intra-articular injection of MIA in WKY rats, plasma levels of β-endorphin were increased compared with levels in saline-treated WKY rats at this timepoint (Fig. 3F, Supplemental Table 4, available at http://links.lww.com/PR9/A130). In our hands, plasma levels of enkephalin were below the detection limits (measured by commercially available ELISA), and therefore, we cannot rule out changes in other endogenous opioids (data not shown).

### 3.5. The spinal cord: a potential site of reduced morphine efficacy in this model of high anxiety and osteoarthritis pain

**Figure 4.**
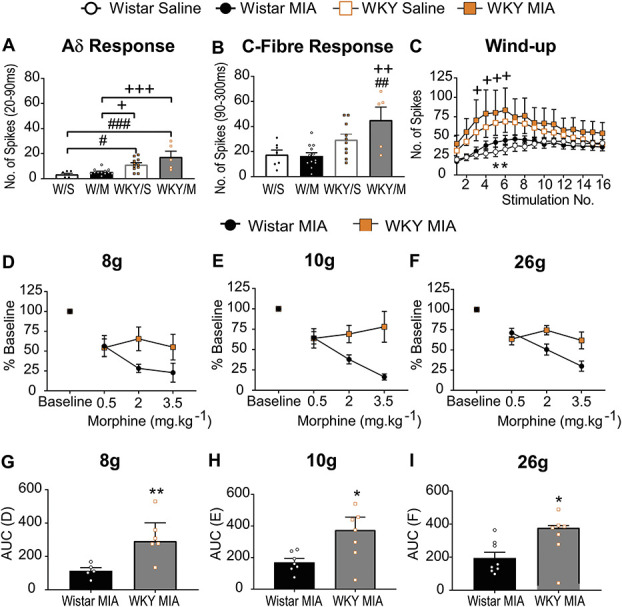
Enhanced excitability of spinal neurons and reduced efficacy of systemic morphine in the WKY-MIA model. *In vivo* electrophysiological recordings of responses of WDR neurons in the deep dorsal horn. The number of action potentials after a noxious electrical stimulus at 3x C-fibre latency was binned according to latency. Responses in the Aδ-fibre latency range were higher in WKY rats compared with Wistar rats (A). Responses in the C-fibre latency range were higher in WKY rats (B) and significantly increased in MIA-treated WKY rats. Data are the average number of action potentials recorded within each poststimulus latency band, with individual data points shown; bars represent the median values and IQR. #*P* = 0.0144, ##*P* = 0.0067, ###*P* = 0.0002 vs Wistar/saline, + *P* = 0.0365, ++ *P* = 0.0017, +++ *P* = 0.0004 vs Wistar/MIA, 2-way ANOVA with the Tukey *post hoc* multiple comparisons test (Supplemental Table 6, available at http://links.lww.com/PR9/A130). Wind-up responses of WDR neurons was greater in WKY rats (C). The number of action potentials recorded in the 90 to 800 ms poststimulus (C fibre to post-stimulus discharge latency range) was significantly higher in saline-injected WKY rats (stimuli 5 and 6) and MIA-injected WKY rats (stimuli 3–6), compared with Wistar rats. Data represent mean ± SEM number of action potentials. *P < 0.05 vs WKY/saline, + *P* < 0.05 vs Wistar/MIA, 2-way ANOVA with the Tukey *post hoc* multiple comparisons test (Supplemental Table 6, available at http://links.lww.com/PR9/A130). There was a blunted effect of 2 and 3.5 mg.kg^−1^ morphine on mechanically evoked responses of spinal WDR neurons in MIA-injected WKY rats: 8 g- (D), 10 g- (E), and 26 g-evoked (F) vFH evoked responses of WDR neurons in MIA-injected WKY rats, which was confirmed by AUC analysis (G–I). Data represent median ± IQR, *P < 0.05, **P < 0.01 Mann–Whitney *U* tests (Supplemental Table 3, available at http://links.lww.com/PR9/A130). ANOVA, analysis of variance; MIA, monosodium iodoacetate; PWTs, paw withdrawal thresholds; vFH, von Frey hair; WDR, wide dynamic range; WKY, Wistar Kyoto.

To evaluate a potential locus for the altered behavioural phenotype in the WKY-MIA model, in vivo single-unit recordings of WDR neurons in the spinal cord dorsal horn were made in saline or MIA-treated rats (see Supplemental Figure 4 for sample data, available at http://links.lww.com/PR9/A130). Basal neuronal characteristics were similar across groups (see Supplemental Table 8, available at http://links.lww.com/PR9/A130). Neuronal responses to an overtly nociceptive electrical stimulus (3x C-fibre threshold, 2ms) were significantly higher in the WKY strain and further elevated in MIA-injected rats (Fig. 4A, B, Supplemental Table 7, available at http://links.lww.com/PR9/A130, *P* < 0.0001). Action potentials recorded in the Aδ range were significantly elevated (3-fold) in WKY rats compared with Wistar rats (Fig. 4A, Supplemental Table 6, available at http://links.lww.com/PR9/A130), and responses in the C-fibre latency range were significantly higher in MIA-injected WKY rats, compared with both saline-treated and MIA-treated Wistar rats (Fig. 4B, Supplemental Table 6, available at http://links.lww.com/PR9/A130). A significant effect of strain was also observed for the degree of wind-up, a proxy of central sensitization^35^ (Fig. 4C, Supplemental Table 6, available at http://links.lww.com/PR9/A130 *P* < 0.0001), with the highest responses evident in MIA-treated WKY rats. There were no differences in responses in the nonnoxious Aβ-fibre latency, post-stimulus discharge (Supplemental Figure 5A&B, available at http://links.lww.com/PR9/A130), or mechanically evoked responses of WDR neurons (Supplemental Figure 5C, Supplemental Table 6, available at http://links.lww.com/PR9/A130).

In line with our behavioural data, inhibitory effects of consecutive systemic doses of morphine on evoked responses of spinal neurons were significantly blunted in MIA-treated WKY rats, compared with Wistar rats (Fig. 4D–I). The lowest dose of morphine (0.5 mg·kg^−1^) had a similar mean maximal inhibitory effect on a range of noxious and nonnoxious mechanical stimuli–evoked responses, but there was little additional inhibition after successive increased doses of morphine in the WKY-MIA rats, unlike inhibitory effects evident in Wistar-MIA rats (Fig. 4D–E, G–H, Supplemental Table 3, available at http://links.lww.com/PR9/A130).

### 3.6. Spinal µ-opioid receptor function altered in the high anxiety model of osteoarthritis pain

**Figure 5.**
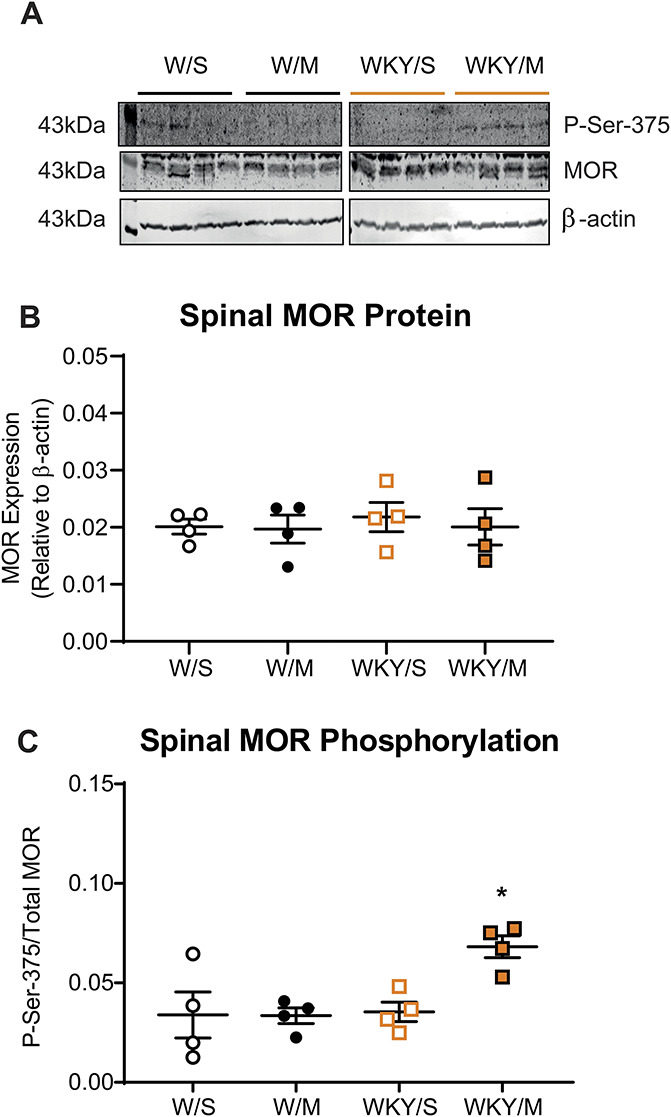
Altered P-ser375 in the spinal cord in the WKY or MIA model. Western blots of the expression of total MOR and P-ser375 in ipsilateral lumbar spinal cord homogenates (A). Densitometry quantification revealed consistent levels of total MOR (B) across strains and treatments (n = 4/group) and in P-ser375 in MIA-injected Wistar rats (C). There was a significant increase in the proportion of P-ser375MOR in MIA-injected WKY rats, compared with saline-injected controls. For expanded images of Western blots, see Supplemental Figure 6, available at http://links.lww.com/PR9/A130). Bars represent median values and IQR, *P = 0.0312, 2-way ANOVA with the Tukey *post hoc* multiple comparisons test (Supplemental Table 6, available at http://links.lww.com/PR9/A130). ANOVA, analysis of variance; MIA, monosodium iodoacetate; MOR, µ-opioid receptors; WKY, Wistar Kyoto.

To explore potential changes in MOR function, we quantified spinal cord MOR expression. Western blotting revealed no significant effect of strain or treatment on total dorsal horn MOR expression (Fig. 5A, B). There was a significant increase in phosphorylation at P-ser375 in MIA-treated WKY rats compared with saline-treated WKY rats (Fig. 5C, Supplemental Table 6, available at http://links.lww.com/PR9/A130). This was not evident in Wistar rats (Fig. 5C, Supplemental Table 6, available at http://links.lww.com/PR9/A130). These data support an alteration in spinal MOR function in MIA-treated WKY rats, in the absence of exogenous morphine treatment.

## 4. Discussion

Clinically, people with high anxiety and chronic pain, including OA pain, have greater opioid consumption compared with people with comparable pain but less anxiety.^8^ This clinical problem was mimicked in a rodent model of high anxiety and OA-like pain; our mechanistic studies revealed multiple lines of evidence supporting altered endogenous opioid function, including increased phosphorylation at serine residue 375 of MOR, which is required for opioid-mediated desensitization,^50^ in the absence of previous exposure to exogenous opioid ligand.

Systemically administered morphine produced a robust inhibition of behavioural pain responses after OA-induced joint pathology in Wistar rats, which do not normally exhibit an anxiogenic phenotype. However, in WKY rats, which have a heightened anxiety-like phenotype, the effects of morphine on OA-induced pain behaviour were significantly blunted. The lowest dose of morphine did not reverse weight-bearing asymmetry or hind paw withdrawal thresholds in MIA-treated WKY rats, despite significant inhibitory effects in the MIA-treated Wistar rats. This may reflect the greater pain behaviour in MIA-treated WKY rats; however, a similar low dose of morphine reversed far greater weight-bearing asymmetry induced by tibio-tarsal carrageenan injection.^49^ In our study, the highest dose of systemic morphine studied produced comparable inhibitory effects on MIA-induced pain behaviour in both strains of rats, thus reduced efficacy of opioid signalling, rather than a loss of receptors in WKY rats, is likely to explain these differences in the effects of morphine. Plasma levels of morphine after systemic administration have been shown to be equivalent in WKY and SD rats,^23^ and therefore, metabolism of exogenous opioids is an unlikely confounder. Reduced efficacy of morphine in WKY rats has been reported in both acute pain tests and the formalin model,^23^ and here, we report blunted opioid analgesia in a clinically relevant model of chronic OA pain in WKY rats. Our data are consistent with the clinical evidence that anxiety in people with joint pain is associated with the greater^8^ or more prolonged^41^ use of prescription opioids.

To investigate the basis for the blunted analgesic efficacy of morphine in WKY rats with the model of OA pain, the effects of pharmacological blockade of MOR on pain behaviour were explored. Systemic naloxone or the MOR-selective antagonist CTAP lowered hind paw withdrawal thresholds in Wistar rats in the presence of the model of OA pain, but not in pain-free, saline-treated Wistar rats. These data are consistent with the engagement of the endogenous opioidergic systems to counter increased pronociceptive signalling in models of chronic pain and align with evidence from clinical pain states.^24,30,34^ Unlike Wistar rats, both systemic naloxone or CTAP significantly lowered hind paw withdrawal thresholds in the absence of the model of OA pain in the WKY strain of rats, suggesting the endogenous opioidergic system is basally active in WKY rats in the absence of a pain state. In the presence of the model of OA pain in WKY rats, neither systemic naloxone nor CTAP had any additional effect on the hind paw withdrawal thresholds, suggesting in the presence of the model of OA pain, the endogenous opioidergic system is not further activated in WKY rats. One plausible explanation for this lack of chronic pain-induced engagement of the opioidergic inhibitory pathways in WKY rats is that the endogenous opioid system is already maximally activated in the absence of the pain state.

Increased levels of β-endorphin have previously been reported in the MIA model of OA pain in mice.^2^ In this article, baseline plasma levels of β-endorphin were significantly lower in WKY rats compared with Wistar rats; this difference was also observed after intra-articular injection of saline in the 2 strains of rats. These data do not neatly align with the effects of naloxone and CTAP, which suggest the presence of greater endogenous opioid tone in WKY rats in the absence of the OA pain model. This may point to a role of other endogenous opioid peptides; however, in our hands, plasma levels of enkephalin were below the limits of the detection assay and could not be tested. In the presence of established MIA-induced OA pain behaviour, only WKY rats had elevated levels of plasma β-endorphin, supporting a specific role in the model of high anxiety and OA pain behaviour. Nevertheless, contributions of other endogenous opioid peptides and the relationship between circulating and tissue-specific levels of opioids^27^ remain to be determined.

The spinal cord dorsal horn is a key signalling hub in pain pathways^54^ and a major site of action of both endogenous and exogenous opioids.^18,59^ Recordings of spinal cord dorsal horn WDR neurons in MIA-treated WKY rats compared with MIA-treated Wistar rats showed that the effects of systemic morphine on WDR responses to low-force and high-force mechanical stimulation of the hind paw were blunted in WKY MIA-treated rats. These effects may arise as a result of altered MOR function or exacerbated pronociceptive signalling due to inflammatory processes^29^ or increased NMDA receptor signalling.^36,52,60^ We hypothesised that these blunted inhibitory effects of exogenous morphine may arise, at least in part, due to desensitization or dysregulation of MOR.

The C terminus of MOR has a number of phosphorylation sites that contribute to receptor desensitization and internalization. Of the residues that undergo agonist-dependent phosphorylation, residues 375 to 379 (STANT) have a critical role in endogenous opioid-induced acute desensitization, recovery from desensitization, and internalization of MOR.^4,32^ Phosphorylation of the serine-375 residue of MOR was significantly elevated in the ipsilateral dorsal horn of the spinal cord of MIA-treated WKY rats, suggesting greater MOR tolerance in MIA-treated WKY rats. This may account, at least in part, for the loss of inhibitory effect of morphine under this condition. Nevertheless, we cannot discount changes in MOR expression or phosphorylation at other sites in the brain.

The limitations of our study include the use of an inbred rat strain to model anxiety-like behaviour. Differences in MOR gene expression between WKY and Sprague–Dawley rats have been reported for some brain regions.^13^ Despite no differences in MOR expression in the reward-associated nucleus accumbens,^22^ acquisition of morphine-induced conditioned place preference was reduced in WKY rats, supporting our evidence for dysfunctional responses to exogenous opioids in this strain. Alongside altered opioid function, WKY rats have lower basal levels of limbic serotonin and dopamine, resulting in a blunted response to acute stress,^21^ which is likely relevant to the clinical situation. Given the key roles for supraspinal monoamines in descending modulation of pain signalling,^7^ it is possible that both opioidergic and monoaminergic dysfunction contribute to augmented OA-like pain responses in WKY rats. Our study was only performed in male rats; although the relationship between anxiety and prescription opioid use for chronic pain is stronger in males than females,^47^ future work in female rats is important. Finally, although MOR internalization was not quantified here, increased MOR internalization in dorsal root ganglia has been demonstrated in a model of chronic inflammatory pain^61^ and shown to be further increased by morphine treatment.

Understanding why people with high anxiety and OA pain have higher opioid consumption is essential to develop alternative treatment strategies. Using a translationally relevant model, our data support a role of altered endogenous opioid receptor function and increased phosphorylation at serine residue 375 of MOR, which is required for morphine-mediated desensitization,^50^ in the absence of previous exposure to exogenous opioid ligand. Our study highlights the functional impact of the combination of anxiety, chronic pain, and altered opioidergic tone, which leads not only to increased pain responses but also to decreased efficacy of opioid analgesia.

## Disclosures

The authors have no conflicts of interest to declare.

## Appendix A. Supplemental digital content

Supplemental digital content associated with this article can be found online at http://links.lww.com/PR9/A130.

## Supplementary Material

SUPPLEMENTARY MATERIAL
