# Supplementary material for: Anxiety enhances pain in a model of osteoarthritis and is associated with altered endogenous opioid function and reduced opioid analgesia
Source: Pain Rep. 2022 Feb 3;6(4):e956. doi: 10.1097/PR9.0000000000000956 (PMC8568395; doi:10.1097/PR9.0000000000000956)
Supplement: SUPPLEMENTARY MATERIAL [file painreports-6-e956-s001.docx]

**Title: Anxiety enhances pain in a model of osteoarthritis and is associated with altered endogenous opioid function and reduced opioid analgesia**

**Abbreviated Title:** Anxiety & opioid analgesia in OA pain

**Supplemental Digital Content**

**Supplemental Table 1 – Animals excluded from study**

| **1. Non-matching cartilage scores** | **Wistar/Saline** | **Wistar/MIA** | **WKY/Saline** | **WKY/MIA** |
| --- | --- | --- | --- | --- |
| **Total No.** | **3** | **4** | **3** | **4** |
| Cohort 2 (N) | 1/12 | 1/12 | 1/10 | 2/11 |
| Cohort 3 (E) | 2/17 | 2/22 | 3/19 | 2/20 |
| **2. Incomplete behavioural data** | **Wistar/Saline** | **Wistar/MIA** | **WKY/Saline** | **WKY/MIA** |
| Cohort 2 (N) | 1/12 | 1/12 | 1/10 | 2/11 |
| Cohort 4 (C) | 0/10 | 0/10 | 1/10 | 0/10 |
| **3. Incomplete electrophysiology data** | **Wistar/Saline** | **Wistar/MIA** | **WKY/Saline** | **WKY/MIA** |
| Cohort 3 (E) | 2/10 | 5/15 | 3/12 | 2/12 |
| **4. Abnormal physiology** | **Wistar/Saline** | **Wistar/MIA** | **WKY/Saline** | **WKY/MIA** |
| Cohort 4 (C) | 0/10 | 2/10 | 0/10 | 0/10 |

**M = morphine study, N = naloxone study, E = electrophysiology, study C = CTAP study**

**1.** A total of 14 rats were excluded from the study on the basis of joint pathology inconsistent with the recorded intra-articular treatment received. The exclusion criteria were any rats with a total cartilage damage score < 6 for MIA-treated groups, or ≥ 6 for saline-treated groups. These discrepancies likely resulted from experimenter error during blinding, or misplacement of intra-articular injection.

**2.** Behavioural data from 4 rats were excluded from the naloxone time course experiment due to environmental noise disruption, preventing the collection of valid behavioural data. Behavioural data from a further 1 rat was detected as an outlier after performing Grubb’s test and excluded from this study. Data was not collected from 1 rat in the CTAP time course experiment due to a physiological abnormality preventing the collection of valid pain behaviour.

**3.** Incomplete electrophysiological datasets were obtained from 12 animals due to loss of the target cell during recordings

**4.** Behavioural data from 2 rats were excluded due to hindlimb physiological abnormalities.

**Supplemental Methods**

**Behavioural Testing**

Rats were habituated to the testing environments (incapacitance tester and von Frey cages) for 1hr on 2 consecutive days. Baseline measurements were taken in the morning prior to treatment (D0).

**Weightbearing asymmetry** - Healthy rats distribute their weight evenly between limbs, and a weight shift onto the contralateral limb indicates pain at rest in the ipsilateral knee joint[12].

**Paw Withdrawal Thresholds (PWT) –** A change in hindpaw withdrawal threshold in an experimental model of OA reflects referred pain at a site distal to the injured knee joint. We have previously reported bilateral lowering of PWTs in the MIA model in the WKY strain, demonstrating a wide-spread pain phenotype mirroring clinical presentation of OA patients with elevated anxiety scores[3].

**Elevated Plus Maze (EPM)** - Rats were placed into the centre of the arena with their nose pointing into an open arm and the centrepoint of the animal tracked for 10 minutes. Some exploratory behaviour in the open arms of the maze is expected in normo-anxiety animals, whilst restriction of activity to the closed arms is considered a surrogate indicator of anxiety-like behaviour.

**Locomotor Activity –** The locomotor activity box measured 39.5cm x 23.5cm x 24.5cm, with a 4 x 8 photobeam array (Photobeam Activity System, San Diego Instruments, USA). To correct for strain differences in total bodyweight, locomotor activity was assessed as the number of beam breaks per minute per kilogram bodyweight.

***In vivo* Spinal Electrophysiology**

Single unit extracellular recordings were made from wide dynamic range (WDR) neurons in the deep dorsal horn, as previously described[[60](#_ENREF_60)]. Briefly, a laminectomy was performed under isoflurane anaesthesia (surgery: 3%, maintenance: 1.5%) to expose lumbar L4-6 spinal cord, and a WDR neurone with a receptive field in the toes of the ipsilateral hindpaw was located via a glass-coated tungsten microelectrode (Wistar/saline *n*=10, Wistar/MIA *n*=15; WKY/saline *n=*12, WKY/MIA *n*=12). Once identified, responses of WDR neurones were characterised via electrical stimuli delivered to the peripheral receptive field via bipolar electrodes. WDRs exhibit responses to electrical stimulation at Aβ, Aδ, and C fibre latencies, and wind up in response to a repeated noxious electrical stimulation (16 x 50ms, 0.5Hz, delivered at 3-fold C fibre threshold). The degree of wind up can be used as a proxy of central sensitization[[22](#_ENREF_22)]. Following wind up, stimulating electrodes were removed and there was a 20min period prior to beginning the mechanical stimuli protocol.

**Β-Endorphin ELISA**

Tail vein blood was collected in heparinised blood collection tubes at baseline and at the end of the study on D21, under brief isoflurane anaesthesia (3% in 1L.min-1 O2) to minimise handling stress. Following blood collection on D21, rats were humanely killed via overdose of sodium pentobarbital (Euthatal, 2mL, i.p.). Samples were centrifuged at 3000rpm for 20mins, and the supernatant plasma collected and stored at -80°C prior to assay. Plasma samples were assayed for β-endorphin in duplicate via a commercially available ELISA kit (Phoenix Pharmaceuticals, Burlingame, CA, USA) according to the manufacturer’s instructions.

**Western Blotting**

Rats were killed via overdose with sodium pentobarbital (Euthatal, 2mL, i.p.), decapitated, and spinal cord tissue rapidly collected via hydraulic extrusion. The lumbar enlargement was hemisected down the midline, snap-frozen in liquid nitrogen, and stored at -80°C until processed. The ipsilateral spinal cord was homogenised in RIPA buffer with protease and phosSTOP inhibitor cocktails (Sigma Aldrich, Gillingham, UK) to prevent degradation and preserve phosphorylation sites. 150µg from each sample was separated via SDS-PAGE, transferred onto nitrocellulose membranes, and probed for expression of total MOR (rabbit anti-mu opioid receptor, Neuromics, RA10104, RRID:AB_2156526 1:500), P-ser375 MOR (rabbit anti-mu opioid receptor Ser375, BIOSS-Stratech, bs-3724R, 1:500), and β-actin (mouse anti-β-actin, Sigma, A5441, 1:5000) via overnight incubation in 5% milk at 4°C. The rabbit polyclonal antibody RA10104 is directed against a 15-amino acid sequence (residues 384‐398) in the C‐terminus of MOR, specificity has been demonstrated via adsorption and omission controls in rat tissue[1; 4]. The rabbit polyclonal antibody bs-3724R is directed against a KLH conjugated synthetic phosphopeptide derived from rat MOR around the highly-conserved phosphorylation site of Ser375 (P-ser-375). In Western blots, bs-3724R produces a strong band at 44kDA in neural tissue from rats[5] and mice[2], and manufacturer control ELISA data revealed high preference for P-ser-375 over MOR[2]. Secondary antibodies were IRDye donkey anti-rabbit 800CW and donkey anti-mouse 680RD (1:5000 in 5% milk, RT, 1.5hr), and resulting fluorescent signal imaged via Licor Odyssey system (LI-COR Biosciences, Cambridge, UK) and resulting bands quantified via densitometry measurements in Image Studio Lite version 5.2 (LI-COR Biosciences). Data are expressed as expression level relative to β-actin.

**Statistical Analyses**

For comparisons between strains, Mann-Whitney *U* tests (**Supplemental Table 3**), unpaired t-tests (Supplemental Table 43) or Wilcoxon signed rank tests (**Supplemental Table** **5**) were used. For datasets with matched values (e.g. pain behaviour time courses) repeated measure 2-way ANOVAs were used (**Supplemental Table 6**), with Dunnett’s post-hoc test for multiple comparisons. All other data were analysed via a repeated measures 2-way ANOVAs (**Supplemental Table 6**), with strain and treatment as the independent variables and Tukey’s post-hoc test for multiple comparisons. Where some datasets had missing values, a Mixed-Effects model analysis was utilised instead (**Supplemental Table 7**). Within-strain comparisons of longitudinal changes in β-endorphin were assessed via unpaired T tests. Data are stated as mean ± standard error of the mean (SEM), or median with interquartile range (IQR), as appropriate.

Detailed statistical information is in **Supplemental Tables 3-7**, grouped via type of statistical comparison. Full experimental data are available from the authors upon request.

**Power calculations for group sizes**

All minimum group sizes were determined via power calculations performed utilising a freely-available online tool:

<http://powerandsamplesize.com/Calculators/Compare-2-Means/2-Sample-Equality>

All data are expressed as means ± standard deviation.

**Behavioural Data:** For detection of differences in pain and anxiety behaviours, data from our previous study comparing WKY and Sprague Dawley (SD) rats in this model were utilised[3]. For anxiety-like behaviour, comparison of time spent in the central zone of the open field maze in seconds for SD = 15 ± 3.68, WKY = 34.2 ± 12.15, effect size 19.20, 80% power, α = 0.05, required minimum sample size = 8.

For pain behaviour, ipsilateral PWTs 21 days after MIA administration were compared, SD = 2.27 ± 2.13, WKY = 5.8 ± 2.20, effect size 3.08, 80% power, α = 0.05, required minimum sample size = 11.

***Ex vivo* analyses:** For detection of differences in plasma β-endorphin levels, and spinal expression of MOR, data from our previous study comparing spinal expression of the astroglial marker glial-fibrillary acidic protein (GFAP) were utilised[3]. Intensity of GFAP labelling 21 days after MIA administration, SD = 7000 ± 65, WKY = 14424 ± 1116, effect size 7424, 80% power, α = 0.05, required minimum sample size = 4.

**Supplemental Table 2 – Summary of animal numbers and behavioural data**

|  | **Wistar/Saline** | **Wistar/MIA** | **WKY/Saline** | **WKY/MIA** |
| --- | --- | --- | --- | --- |
| **Total No.** | **47** | **54** | **47** | **51** |
| Cohort 1 (M) | 8 | 10 | 8 | 10 |
| Cohort 2 (N) | 12 | 12 | 10 | 11 |
| Cohort 3 (E) | 17 | 22 | 19 | 20 |
| Cohort 4 (C) | 10 | 10 | 10 | 10 |
| **Bodyweight (Cohort 3, g, mean ± SD)** | | | | |
| **Basal** | **183±15** | **184±14** | **157±14** ^^^^ | **160±16** ^^^^ |
| **Day 21** | **340±23** | **337±28** | **251±15** ^^^^ | **247±12** ^^^^ |
| **Pain** | | | | |
| **Basal WB %** | **50.16** | **49.75** | **50.42** | **50.13** |
| Cohort 1 (M) | 49.69 | 49.67 | 50.74 | 50.54 |
| Cohort 2 (N) | 50.39 | 48.74 | 50.94 | 50.04 |
| Cohort 3 (E) | 50.23 | 50.37 | 49.98 | 49.95 |
|  | | | | |
| **Day21 WB %** | **48.89** | **36.84** #### | **50.11** | **43.12** ****, + |
| Cohort 1 (M) | 50.08 | 38.99 | 50.99 | 43.97 |
| Cohort 2 (N) | 48.36 | 38.24 | 49.59 | 44.71 |
| Cohort 3 (E) | 48.64 | 34.90 | 49.96 | 41.85 |
|  | | | | |
| **Basal ipsilateral PWT (g)** | **22.12±5.35** | **23.35±5.21** | **20.39±6.42** | **19.78±6.12** |
| Cohort 1 (M) | 21.88±5.69 | 23.30±5.81 | 19.13±5.69 | 18.90±6.30 |
| Cohort 2 (N) | 23.00±5.14 | 26.00±0 | 18.00±8.55 | 20.50±5.88 |
| Cohort 3 (E) | 21.60±5.58 | 22.26±5.75 | 22.12±5.42 | 19.94±6.41 |
|  | | | | |
| **D21 ipsilateral PWT (g)** | **19.47** | **12.71** | **12.69** | **7.17** |
| Cohort 1 (M) | 22.38±6.97 | 15.70±5.89 | 13.63±3.89 | 8.00±6.46 |
| Cohort 2 (N) | 20.09±7.01 | 14.44±8.76 | 17.14±9.35 | 10.25±3.24 |
| Cohort 3 (E) | 17.47±7.60 | 10.32±5.15 | 10.41±4.15 | 5.33±3.82 |
|  | | | | |
| **Mean Δ ipsilateral PWT (vFH)** | **-0.44** | **-1.68**  # | **-1.44**  # | **-3.22**  ####**, *,** ++ |
| Cohort 1 (M) | -0.13±0.99 | -0.88±0.99 | -0.88±1.73 | -2.90±1.29 |
| Cohort 2 (N) | -0.36±1.03 | -1.78±1.48 | -0.57±1.81 | -1.76±1.16 |
| Cohort 3 (E) | -0.67±1.18 | -2.11±1.45 | -2.06±1.34 | -4.06±2.15 |
|  | | | | |
| **Basal contralateral PWT (g)** | **22.29** | **24.31** | **21.33** | **20.95** |
| Cohort 1 (M) | 21.25±6.73 | 26.00±0.00 | 21.88±5.69 | 21.11±5.80 |
| Cohort 2 (N) | 23.00±5.14 | 26.00±0.00 | 19.75±8.65 | 21.88±5.69 |
| Cohort 3 (E) | 22.33±5.37 | 22.53±5.25 | 21.82±5.94 | 20.50±5.64 |
|  | | | | |
| **Day 21 contralateral PWT (g)** | **23.41** | **24.08** | **13.18** | **9.68** |
| Cohort 1 (M) | 24.63±3.89 | 26.00±0.00 | 15.75±5.25 | 8.56±2.70 |
| Cohort 2 (N) | 23.00±5.14 | 23.00±5.14 | 14.63±8.18 | 10.50±6.48 |
| Cohort 3 (E) | 23.07±5.04 | 23.68±4.61 | 11.29±3.02 | 9.85±4.89 |
|  | | | | |
| **Mean Δ contralateral PWT (vFH)** | **0.12** | **-0.03**  #### | **-1.21**  ####, ++++ | **-2.06**  ####, ++++ |
| Cohort 1 (M) | 0.38±0.92 | 0.00±0.00 | -1.00±1.41 | -2.20±1.03 |
| Cohort 2 (N) | 0.00±0.77 | -0.30±0.48 | -0.88±1.13 | -1.75±1.16 |
| Cohort 3 (E) | 0.07±0.70 | 0.11±0.46 | -1.47±0.80 | -2.11±1.18 |
|  | | | | |
| **Basal Anxiety (open arm AUC)** | | | | |
| Cohort 3 (E) | 33.60  (19.20-58.87) | | 17.40  (4.92-31.68)  ^^ | |
| **Anxiety Post-Model Induction (open arm AUC)** | | | | |
| Cohort 3 (E) | 16.56  (4.40-24.84) | 32.16  (15.46-50.61) | 1.38  (0.03-3.96)  ##, +++ | 2.58  (0.12-14.40)  ++ |

Data from the 4 cohorts of animals utilised in this study (M = morphine study, N = naloxone study, E = electrophysiology study, C = CTAP study). Data are expressed as mean ± SD, or median (IQR). Significance assessed via 2-way ANOVA with Tukey multiple comparison post-hoc testing, Kruskal-Wallis test with Dunn’s multiple comparison post-hoc testing, or Mann-Whitney U tests as appropriate.

^^ p<0.01, ^^^^ p<0.0001 versus Wistar/saline & Wistar/MIA

# p<0.05, #### p<0.0001 versus Wistar/saline

* p<0.05, **** p<0.0001 versus WKY/saline

+ p<0.05, ++ p<0.01, +++ p<0.001, ++++ p<0.0001 versus Wistar/MIA.

**Supplemental Table 3 – Statistical Analyses: Mann-Whitney *U* tests**

| **Figure** | **Measurement** | **Mann-Whitney *U*** | **Tails** | **Medians & IQR** | **Difference** | **P Value** |
| --- | --- | --- | --- | --- | --- | --- |
| 1A | Anxiety, duration in open arms (s) | 196 | 1 | Wistar = 35.64, IQR 22.02 - 66.42, *n*=28;  WKY = 15.96, IQR 6.30 – 37.50, *n*=24 | 19.68 | P = 0.0047 |
| 1B | Anxiety, latency to enter open outer arm (s) | 221.5 | 1 | Wistar = 298.8, IQR 86.34 - 600, *n*=28;  WKY = 600, IQR 600 - 600, *n*=24 | 301.2 | P = 0.0077 |
| 3E | Plasma β-endorphin levels (ng.mL^-1^) | 40.5 | 2 | Wistar = 1.00,  IQR 0.90 – 1.08, *n*=18;  WKY = 0.79,  IQR 0.73 – 0.85, *n*=18 | 40.50 | P < 0.0001 |
| 4G | MMI of morphine on 8g-evoked neuronal responses (AUC) | 2 | 1 | Wistar MIA = 111, IQR 79 -152, *n*=5;  WKY MIA = 293, IQR 240 - 402, *n*=6 | 182 | P = 0.0087 |
| 4H | MMI of morphine on 10g-evoked neuronal responses (AUC) | 9 | 1 | Wistar MIA = 160, IQR 130 - 241, *n*=7;  WKY MIA = 375, IQR 233 - 456, *n*=7 | 215 | P = 0.0265 |
| 4I | MMI of morphine on 26g-evoked neuronal responses (AUC) | 11 | 1 | Wistar MIA = 148, IQR 121 - 283, *n*=8;  WKY MIA = 378, IQR 278 - 392, *n*=7 | 230 | P = 0.027 |

**Supplemental Table 4 – Statistical Analyses: Unpaired t-test**

| **Figure** | **Measurement** | ***t, df*** | **Tails** | **Mean & SEM** | **Difference** | **P Value** |
| --- | --- | --- | --- | --- | --- | --- |
| 3F | Δ β-Endorphin (% baseline) | 2.20, 15 | 2 | WKY/S = 116, 3.36, *n*=9;  WKY/M = 128, 4.64, *n*=8 | 12.41 ± 5.64 | P = 0.044 |
| S2B | Locomotor activity 60-90mins after morphine in naïve WKY | 0.59, 9 | 1 | WKY/Saline = 293, 65, *n*=6;  WKY/Morphine =352, 76, *n*=5 | 59.05 ± 99.65 | P = 0.28 |

**Supplemental Table 5 – Statistical Analyses: Wilcoxon signed ranks test**

| **Figure** | **Measurement** | **Group** | ***n*** | **Theoretical Median** | **Actual Medians (0.5, 2.5, 6mg.kg^-1^)** | | | **Sum of Signed Ranks**  **(0.5, 2.5, 6mg.kg^-1^)** | | | **P Values** |
| --- | --- | --- | --- | --- | --- | --- | --- | --- | --- | --- | --- |
| 2A | Behavioural response to morphine, % analgesia (weightbearing) | Wistar/MIA | 10 | 0 | 39.85 | 69.84 | 87.05 | 55 | 55 | 55 | All 0.002 |
|  |  | WKY/MIA | 10 | 0 | -3.65 | 35.56 | 78.04 | -1 | 35 | 51 | >0.999, 0.084, 0.006 |

**Supplemental Table 6 – Statistical Analyses: Two-way ANOVAs, including repeated measures**

**RM** indicates repeated measures design

| **Figure** | **Measurement** | **Source of Variation** | **% Total Variation** | **DF** | **F (DFn, DFd)** | **P value** | ***Post-hoc* test** |
| --- | --- | --- | --- | --- | --- | --- | --- |
| 1E | Anxiety, duration in open arms (s) | Interaction | 0.49 | 1 | F (1, 48) = 0.3580 | P=0.5524 | Tukey, compare all group means |
|  |  | Treatment | 4.21 | 1 | F (1, 48) = 3.068 | P=0.0862 |  |
|  |  | Strain | 30.26 | 1 | F (1, 48) = 22.07 | P<0.0001 |  |
| 1F | Joint pathology, combined cartilage damage score | Interaction | 0.00 | 1 | F (1, 80) = 0.00009 | P=0.9924 | Tukey, compare all group means |
|  |  | Treatment | 81.86 | 1 | F (1, 80) = 588.0 | P<0.0001 |  |
|  |  | Strain | 2.98 | 1 | F (1, 80) = 21.38 | P<0.0001 |  |
| 2B  **RM** | Behavioural response to morphine, change in ipsilateral PWT (log vFH) | Interaction  (Dose x Strain) | 5.86 | 4 | F (4, 72) = 3.720 | P=0.0083 | Dunnett, compare group means within strain/ treatment at each dose |
|  |  | Dose | 20.96 | 4 | F (3.248, 58.47) = 13.30 | P<0.0001 |  |
|  |  | Strain | 36.22 | 1 | F (1, 18) = 76.08 | P<0.0001 |  |
|  |  | Animal | 8.57 | 18 | F (18, 72) = 1.208 | P=0.2784 |  |
| 2C  **RM** | Behavioural response to morphine, change in contralateral PWT (log vFH) | Interaction  (Dose x Strain) | 9.70 | 4 | F (4, 72) = 12.66 | P<0.0001 | Dunnett, compare group means within strain/ treatment at each dose |
|  |  | Dose | 9.70 | 4 | F (3.202, 57.64) = 12.66 | P<0.0001 |  |
|  |  | Strain | 54.60 | 1 | F (1, 18) = 80.37 | P<0.0001 |  |
|  |  | Animal | 12.23 | 18 | F (18, 72) = 3.550 | P<0.0001 |  |
| 3A, **RM** | Behavioural response to naloxone, change in ipsilateral PWT (log vFH) | Interaction  (Dose x Strain/Treatment) | 5.03 | 9 | F (9, 87) = 3.310 | 0.0016 | Tukey, compare group means between strain/ treatments at each dose |
|  |  | Dose | 5.76 | 3 | F (2.219, 64.35) = 11.38 | P<0.0001 |  |
|  |  | Strain/Treatment | 47.64 | 3 | F (3, 29) = 16.30 | P<0.0001 |  |
|  |  | Animal | 28.25 | 29 | F (29, 87) = 5.774 | P<0.0001 |  |
| 3B, **RM** | Behavioural response to naloxone, change in contralateral PWT (log vFH) | Interaction  (Dose x Strain/Treatment) | 4.29 | 9 | F (9, 87) = 3.183 | 0.0023 | Tukey, compare group means between strain/ treatments at each dose |
|  |  | Dose | 6.19 | 3 | F (2.612, 75.75) = 13.76 | P<0.0001 |  |
|  |  | Strain/Treatment | 53.46 | 3 | F (3, 29) = 21.51 | P<0.0001 |  |
|  |  | Animal | 24.03 | 29 | F (29, 87) = 5.529 | P<0.0001 |  |
| 3C | Behavioural response to naloxone, AUC of ipsilateral PWT dose/response curve (A.U.) | Interaction | 2.48 | 1 | F (1, 29) = 2.447 | 0.1286 | Tukey, compare all group means |
|  |  | Treatment | 7.02 | 1 | F (1, 29) = 6.918 | 0.0135 |  |
|  |  | Strain | 54.05 | 1 | F (1, 29) = 53.28 | P<0.0001 |  |
| 3D | Behavioural response to naloxone, AUC of contralateral PWT dose/response curve (A.U.) | Interaction | 0.01 | 1 | F (1, 29) = 0.01277 | 0.9108 | Tukey, compare all group means |
|  |  | Treatment | 0.21 | 1 | F (1, 29) = 0.2065 | 0.6529 |  |
|  |  | Strain | 69.69 | 1 | F (1, 29) = 67.82 | P<0.0001 |  |
| 4A | No. of Aδ latency action potentials in response to noxious electrical stimulus | Interaction | 2.34 | 1 | F (1, 31) = 1.466 | 0.2352 | Tukey, compare all group means |
|  |  | Treatment | 8.12 | 1 | F (1, 31) = 5.081 | 0.0314 |  |
|  |  | Strain | 49.48 | 1 | F (1, 31) = 30.97 | P<0.0001 |  |
| 4B | No. of C latency action potentials in response to noxious electrical stimulus | Interaction | 6.15 | 1 | F (1, 30) = 3.075 | 0.0897 | Tukey, compare all group means |
|  |  | Treatment | 4.51 | 1 | F (1, 30) = 2.251 | 0.1440 |  |
|  |  | Strain | 35.85 | 1 | F (1, 30) = 17.92 | 0.0002 |  |
| 4C | No. of action potentials in response to wind-up protocol | Interaction | 3.12 | 45 | F (45, 496) = 0.4568 | 0.9991 | Tukey, compare all group means at each stimulus |
|  |  | Stimulus | 6.53 | 15 | F (15, 496) = 2.865 | 0.0002 |  |
|  |  | Strain/treatment | 14.37 | 3 | F (3, 496) = 31.54 | P <0.0001 |  |
| 5B | Spinal expression of MOR, densitometry (A.U.) | Interaction | 0.55 | 1 | F (1, 12) = 0.06900 | 0.7973 | Tukey, compare all group means |
|  |  | Treatment | 1.53 | 1 | F (1, 12) = 0.1907 | 0.6701 |  |
|  |  | Strain | 1.41 | 1 | F (1, 12) = 0.1759 | 0.6824 |  |
| 5C | Ratio of spinal expression of P-ser375-MOR/total MOR, relative densitometry (A.U.) | Interaction | 18.59 | 1 | F (1, 12) = 5.371 | 0.0389 | Tukey, compare all group means |
|  |  | Treatment | 17.75 | 1 | F (1, 12) = 5.128 | 0.0429 |  |
|  |  | Strain | 22.11 | 1 | F (1, 12) = 6.387 | 0.0266 |  |
| S1C | Comparison of strain locomotor activity | Interaction | 1.087 | 1 | F (1, 17) = 0.7075 | 0.412 | Sidak compare group means for each strain at each time point |
|  |  | Time | 19.39 | 1 | F (1, 17) = 0.12362 | 0.0024 |  |
|  |  | Strain | 0.3709 | 1 | F (1, 17) = 0.1235 | 0.7295 |  |
| S2A | Effects of morphine on locomotor activity in WKY | Interaction | 11.26 | 29 | F (29, 261) = 1.959 | P=0.0033 | Sidak compare group means for each treatment at each time point |
|  |  | Time | 20.56 | 29 | F (5.667, 51) = 3.577 | P=0.057 |  |
|  |  | Treatment | 0.45 | 1 | F (1,9) = 0.2842 | P=0.4451 |  |
| S3A | Behavioural response to CTAP, AUC of ipsilateral PWT dose/response curve (A.U.) | Interaction | 2.94 | 1 | F (1, 33) = 3.505 | P=0.0701 | Sidak, compare group means for each strain and treatment |
|  |  | Treatment | 23.29 | 1 | F (1, 33) = 27.78 | P<0.0001 |  |
|  |  | Strain | 40.39 | 1 | F (1, 33) = 48.18 | P<0.0001 |  |
| S3B | Behavioural response to CTAP, AUC of contralateral PWT dose/response curve (A.U.) | Interaction | 1.901 | 1 | F (1, 33) = 2.354 | 0.1345 | Sidak, compare group means for each strain and treatment |
|  |  | Treatment | 0.001808 | 1 | F (1, 33) = 0.002229 | 0.9626 |  |
|  |  | Strain | 70.04 | 1 | F (1, 33) = 86.34 | <0.0001 |  |
| S4A | No. of Aβ latency action potentials in response to noxious electrical stimulus | Interaction | 3.12 | 1 | F (1, 30) = 1.092 | 0.3044 | Tukey, compare all group means |
|  |  | Treatment | 1.20 | 1 | F (1, 30) = 0.4191 | 0.5223 |  |
|  |  | Strain | 6.93 | 1 | F (1, 30) = 2.422 | 0.1301 |  |
| S4B | No. of PD latency action potentials in response to noxious electrical stimulus | Interaction | 0.005 | 1 | F (1, 30) = 0.001529 | 0.9691 | Tukey, compare all group means |
|  |  | Treatment | 4.25 | 1 | F (1, 30) = 1.371 | 0.2509 |  |
|  |  | Strain | 0.87 | 1 | F (1, 30) = 0.2797 | 0.6008 |  |
| S4C, **RM** | No. of action potentials in response to mechanical stimulation | Interaction  (Stimulus force vFH x Strain/Treatment) | 0.68 | 9 | F (9, 102) = 0.6402 | 0.7602 | Tukey, compare group means for each Strain/Treatment at each vFH |
|  |  | Stimulus force (vFH) | 48.82 | 3 | F (1.621, 55.12) = 138.1 | P <0.0001 |  |
|  |  | Strain/Treatment | 0.28 | 3 | F (3, 34) = 0.09690 | 0.9612 |  |
|  |  | Subject | 32.88 | 34 | F (34, 102) = 8.206 | P <0.0001 |  |

**Supplemental Table 7 – Statistical Analyses: Mixed Effects Model ANOVAs**

| **Figure** | **Measurement** | **Fixed Effects** | **F (DFn, DFd)** | **P value** | **Post-hoc test** | **Comparison** | **P Value** |
| --- | --- | --- | --- | --- | --- | --- | --- |
| 1C | Change in ipsilateral PWT (no. of vFH) | Time | F (4.437, 553.1) = 37.36 | P < 0.0001 | Tukey, compare means between each group at each time point | Wistar Saline, Wistar MIA | D10: P = 0.0014;  D17: P < 0.0001,  D21: P = 0.0004 |
|  |  | Strain / Treatment | F (3, 137) = 24.28 | P < 0.0001 |  | WKY Saline, WKY MIA | D10: P = 0.0016;  D17: P = 0.028,  D21: P = 0.0007 |
|  |  | Interaction (Time x Strain / Treatment) | F (18, 748) = 4.956 | P < 0.0001 |  |  |  |
| 1D | Change in contralateral PWT (no. of vFH) | Time | F (4.792, 599.8) = 30.85 | P < 0.0001 | Tukey, compare means between each group at each time point | Wistar Saline, Wistar MIA | ns |
|  |  | Strain / Treatment | F (3, 138) = 44.79 | P < 0.0001 |  | WKY Saline, WKY MIA | D10: P = 0.0009;  D17: P < 0.0034,  D21: P = 0.0103 |
|  |  | Interaction (Time x Strain / Treatment) | F (18, 751) = 14.49 | P < 0.0001 |  |  |  |
| S1A  **RM** | Behavioural response in the MIA-model, % weight bearing asymmetry | Time | F (4.522, 577.3) = 41.76 | P < 0.0001 | Tukey, compare means between each group at each time point | Wistar Saline, Wistar MIA | D3, D7, D10, D14, D17 and D21: all P < 0.0001. |
|  |  | Strain/Treatment | F (3, 140) = 117.7 | P < 0.0001 |  | WKY Saline, WKY MIA | D3, D10, D14, D17 and D21: all P < 0.0001.  D7: P = 0.0002 |
|  |  | Interaction (Time x Strain/ Treatment) | F (18, 766) = 11.62 | P < 0.0001 |  | Wistar MIA, WKY MIA | D10: P = 0.054;  D14: P = 0.0001;  D17: P = 0.031,  D21: P = 0.0011. |
| S1B  **RM** | Bodyweight differences between strain and in the MIA-model | Time | F (1.491, 106.8) = 3343 | P < 0.0001 | Tukey, compare all group means | Wistar Saline, Wistar MIA | ns |
|  |  | Strain/Treatment | F (3, 76) = 77.63 | P < 0.0001 | Tukey, compare all group means | WKY Saline, WKY MIA | ns |
|  |  | Interaction (Time x Strain/ Treatment) | F (9, 215) = 80.61 | P < 0.0001 | Tukey, compare all group means |  |  |

Statistical information on Mann-Whitney *U* tests (**Table 3**), unpaired t-tests (**Table 4**), Wilcoxon signed ranks tests (**Table 5**), 2 way ANOVAs, including repeated measures (**Table 6**), and Mixed -Effects model ANOVAs (**Table 7**). Exact values are given for all incidences where P>0.0001. P values for *post-hoc* multiple comparison tests can be found in the appropriate figure legend.

**Supplemental Results**

**Effects of MOR-specific antagonist CTAP on OA-like pain in the MIA model**

Pain behaviour following consecutive administration of the selective μ-opioid antagonist CTAP (0.1, 0.3, 1mg.kg.mL^-1^, i.p., 60 mins) at 21 days after intra-articular saline or MIA injection in Wistar and WKY rats. CTAP did not alter ipsilateral PWTs in saline-treated Wistar rats (log PWT 1.189 versus 1.114), but significantly lowered ipsilateral PWTs in MIA-treated Wistar rats (log PWT 0.9259 versus 0.7654, p=0.039). In WKY rats, ipsilateral PWTs were lowered in the presence of CTAP, irrespective of treatment with saline (log PWT 0.8753 versus 0.7195, p=0.0006) or MIA (log PWT 0.7730 versus 0.5947, p=0.0057). CTAP did not alter contralateral PWTs in Wistar rats 21 days after intra-articular injection with saline (log PWT 1.147 versus 1.096) or MIA (log PWT 1.110 versus 1.004). However, contralateral PWTs were lowered in the presence of CTAP in WKY rats treated with saline (log PWT 0.8753 versus 0.6999, p= 0.0004) or MIA (log PWT 0.8781 versus 0.7378, p= 0.0014). Data not shown. See **Supplemental Figure 3** for AUC analyses of these data.

**Supplemental Figure 1 – Weight bearing asymmetry in the MIA-model & strain differences in bodyweight**


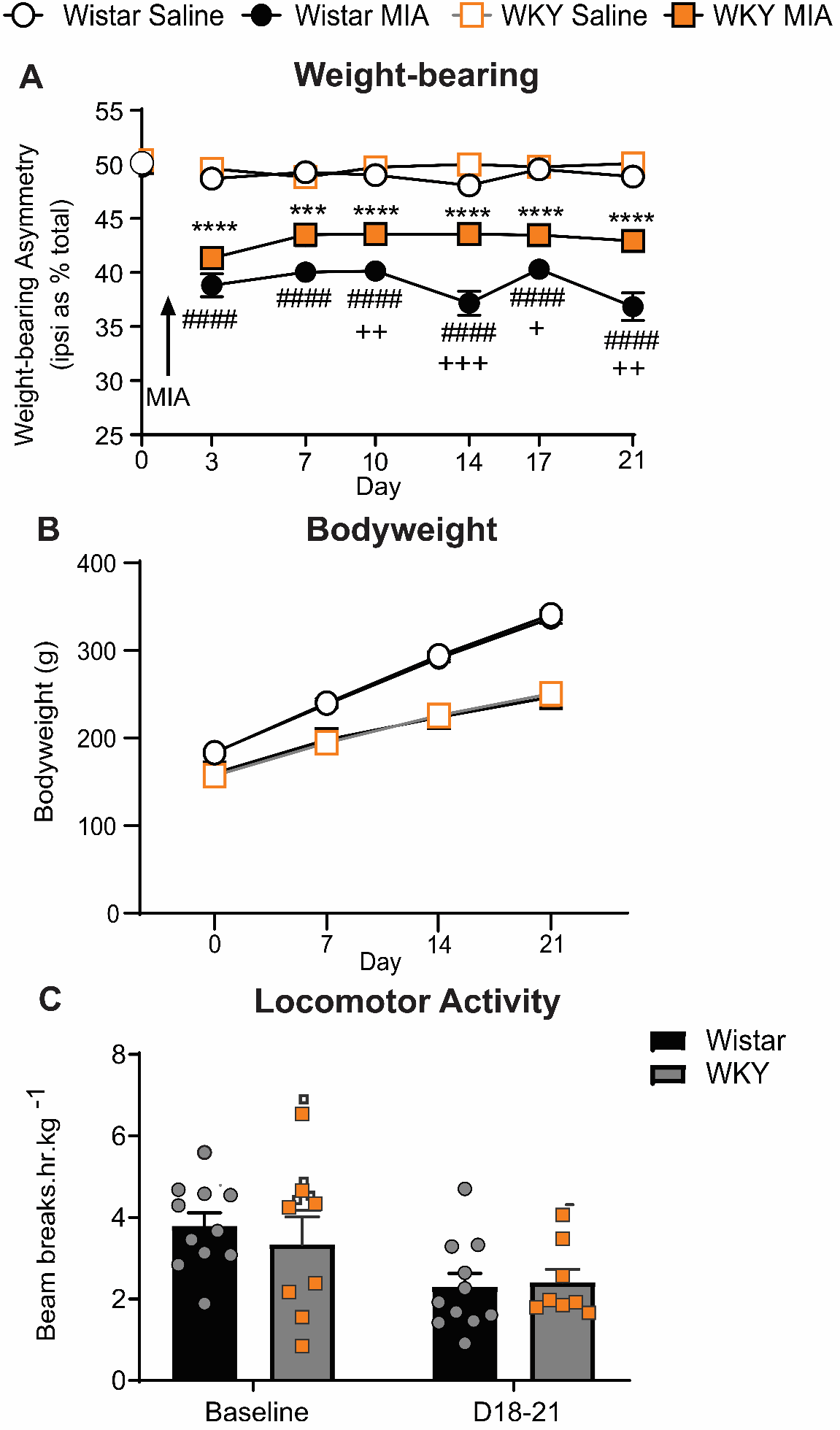


MIA-treated rats developed a similar degree of weightbearing asymmetry in both the Wistar and WKY strains (**A**), with pain behaviour evident from day 3 onwards and maintained until post-injection day 21 (W/S *n*=34, W/M *n*=40, WKY/S *n*=34, WKY/M *n*=36). The slightly smaller magnitude of effect of MIA treatment in the WKY strain is likely due to the presence of a contralateral pain phenotype in this strain. Saline administration did not affect weightbearing in either strain. Data are mean ± SEM % weight borne on the ipsilateral hindlimb, #### p< 0.001 versus Wistar saline, *** p<0.001, **** p<0.0001 versus WKY saline, + p<0.05, ++ p<0.01, +++ p<0.001 versus WKY MIA, Mixed-Effects model with Tukey’s post-hoc mutiple comparison test (**Supplemental** **Table 7**).

Comparison of bodyweights during the study revealed no effect of MIA treatment on bodyweight within strains (**B**), but WKY rats were significantly smaller than Wistar rats at all time points (W/S *n*=18, W/M *n*=21, WKY/S *n* = 22, WKY/M *n*=19). p<0.0001 for all study days, mixed-effects model with Tukey’s *post-hoc* mutiple comparison test (**Supplemental Table 7**).

To ensure any behavioural differences observed in the EPM did not result from strain differences in locomotion, locomotor activity was assessed over a 1 hour period at baseline, and 18-21 days after model induction in a subset of rats (**C**). No significant differences between strains were observed at either time point point (Wistar *n*=11, WKY *n*=8). Data are expressed as mean ± SEM beam breaks per hour, adjusted for bodyweight. Repeated measures 2-way ANOVA with Sidak’s *post-hoc* multiple comparison test (**Supplemental Table 6**).

**Supplemental Table 8 – WDR neuron characteristics**

|  | **Depth (µm)** | **Aβ Fibre Threshold (mA)** | **Aβ Fibre Latency (ms)** | **C Fibre Threshold (mA)** | **C-Fibre Latency (ms)** |
| --- | --- | --- | --- | --- | --- |
| **Wistar Saline** | 770  (630 – 873) | 0.13  (0.11 – 0.15) | 9  (6 - 12) | 1.00  (0.80 – 1.38) | 184  (144 - 243) |
| **Wistar MIA** | 775  (650 – 893) | 0.14  (0.11 – 0.15) | 11  (7 - 12) | 1.00  (0.90 – 1.10) | 205  (167 - 241) |
| **WKY Saline** | 845  (630 - 980) | 0.10  (0.09 – 0.14) | 6  (4 - 12) | 1.00  (0.90 – 1.10) | 144  (108 - 195) |
| **WKY MIA** | 780  (745 - 805) | 0.11  (0.09 – 0.13) | 6  (6 – 11) | 1.00  (0.88 – 1.13) | 223  (157 – 271) |

No significant differences in the depth, Aβ or C-Fibre thresholds or latencies between experimental groups in this study. There was a slight trend towards decreased Aβ latencies and thresholds in the WKY strain, and increased C-fibre latencies for neurones recorded from MIA-treated rats of either strain when compared to saline-treated controls.

Data are median values with IQR. Statistical comparisons via 2 way ANOVA with Tukey’s multiple comparison *post-hoc* testing.

**Supplemental Figure 2 – Effects of morphine on locomotor activity in naive WKY rats**

**
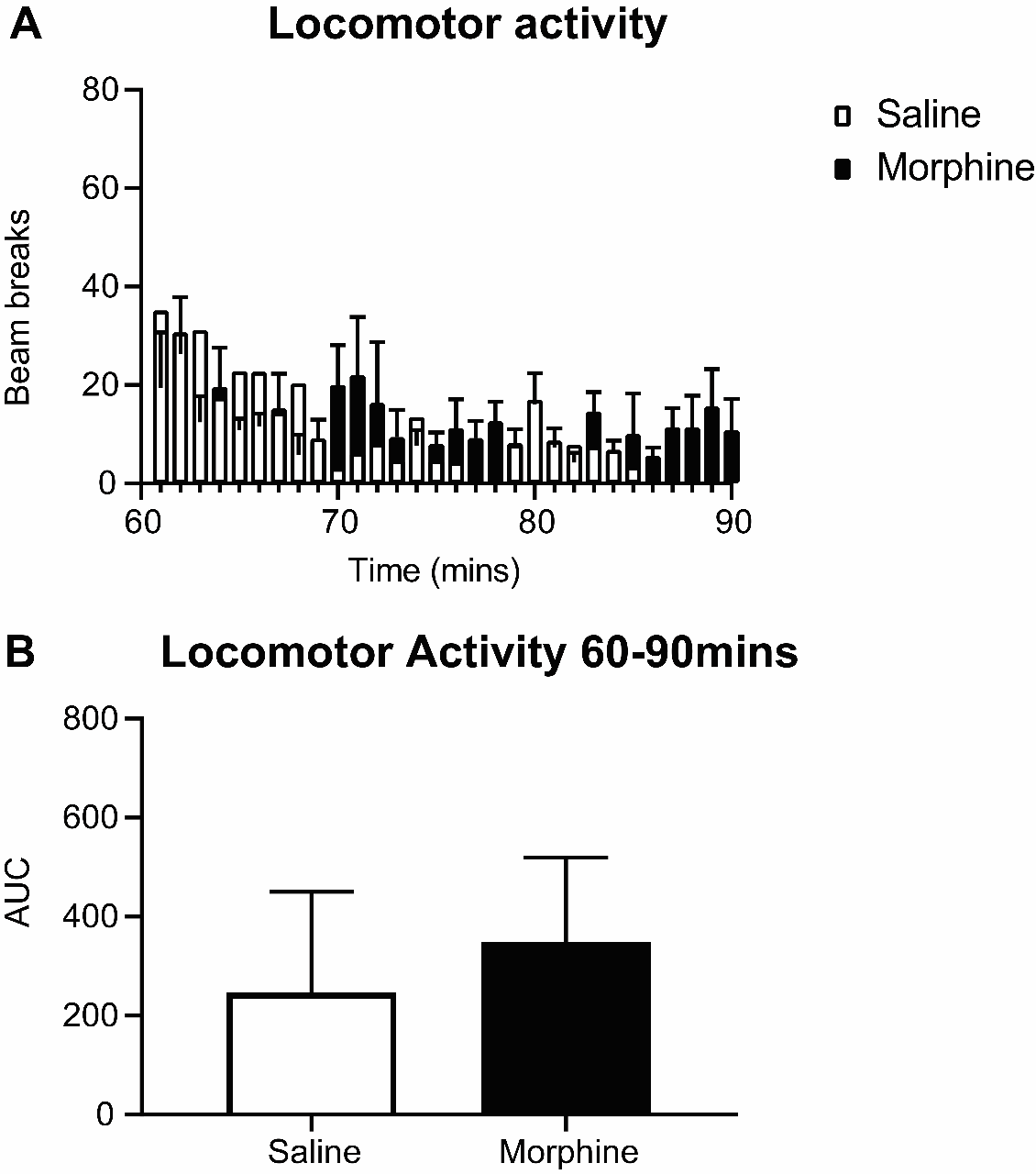
**

Locomotor activity assessed 60-90 mins after the last of 3 consecutive doses of morphine (0.5, 2, & 3.5mg.kg.mL^-1^, s.c.; *n*=6) or saline (50µl; *n*=6) in naïve WKY rats. No differences in locomotor activity between strains were observed after morphine administration. **A:** Locomotor activity assessed as total number of beam breaks per minute. Data represent mean ± SEM. No significant effect of treatment, p=0.6079, repeated measures 2-way ANOVA with Sidak’s *post-hoc* multiple comparison test (**Supplemental Table 6**). **B:** AUC of beam breaks during 60-90 min after morphine injections. Data are mean ± SEM, no effect of treatment, p=0.284 one-tailed unpaired t-test (**Supplemental Table 4**).

**Supplemental Figure 3 - Behavioural responses following CTAP**

**
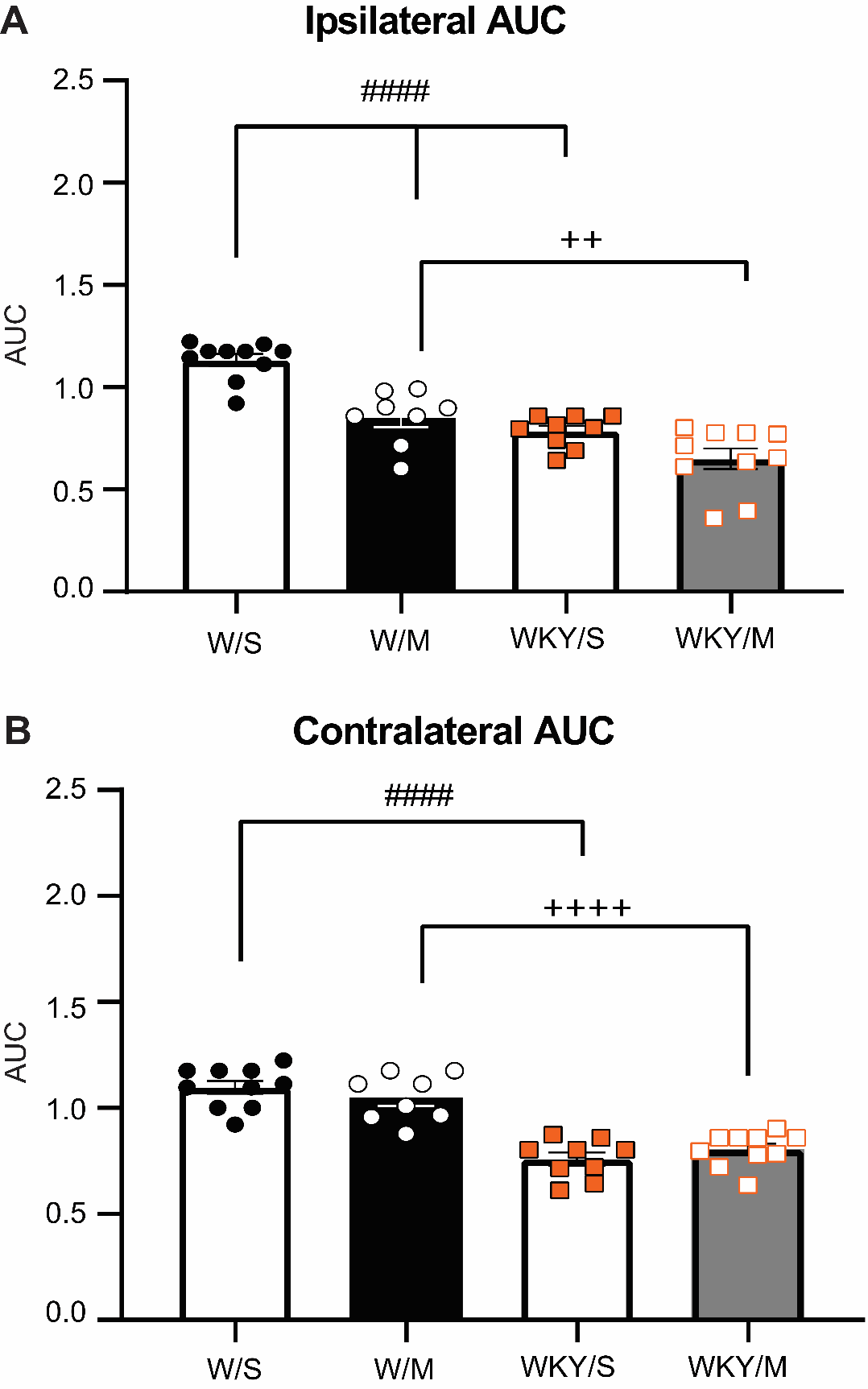
**

**A:** Area under the curve analyses of dose response to cumulative dosing with CTAP (0.1, 0.3, 1mg.kg.mL^-1^, i.p., 60 mins) 21 days after saline or MIA administration reveals a significantly greater effect on ipsilateral PWT in MIA-treated Wistar rats compared to those treated with saline. Blockade of MOR via CTAP had a significantly greater effect in WKY rats when compared to their respective groups of Wistar rats (**Supplemental Table 6**).

**B**: Area under the curve analyses of dose response to cumulative dosing with CTAP on contralateral PWTs reveals no significant differences between Wistar rats treated with saline or MIA, but a significantly greater effect of CTAP in WKY rats (**Supplemental Table 6**)**.**

Data represent area under the curve analysis of dose/response curves. Individual data points are shown with bars representing mean values and error bars depicting SEM. #### p<0.0001 versus Wistar saline; ++ p<0.01, ++++ p<0.0001 versus Wistar MIA. 1-way ANOVA with Sidak’s multiple comparison *post-hoc* testing.

**Supplemental Figure 4 - Spinal neuronal responses to somatosensory input**

**
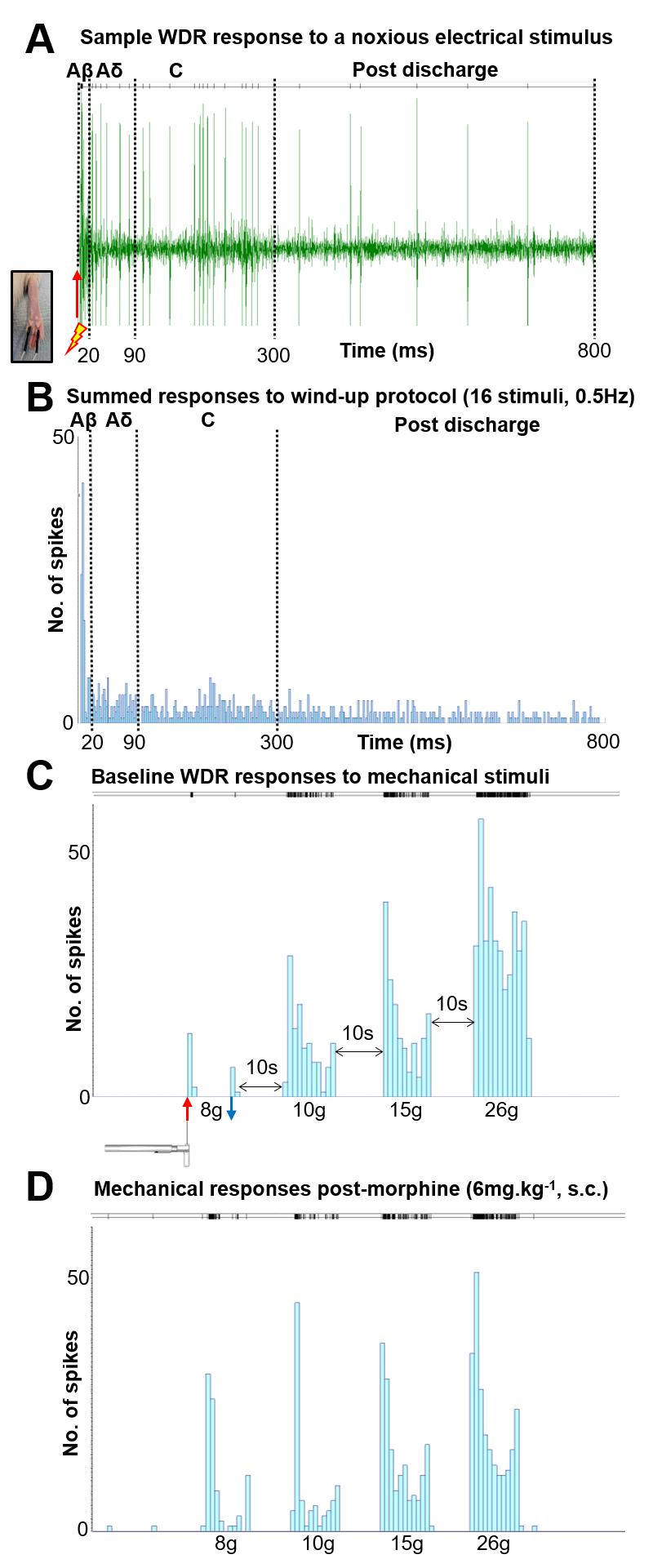
**

**A** – Raw trace showing responses of a WDR cell to a single electrical stimulus delivered to the hindpaw at 3 x C fibre threshold. Dotted lines represent the bins corresponding to the latencies of the main afferent fibres types: Aβ (0-20), Aδ (20-90), & C fibres (90-300ms), and post-discharge (300-800ms). **Red** arrow marks time the stimulus was delivered.

**B** – Histogram displaying the summed responses of the same WDR neuron to the wind-up protocol (train of 16 electrical stimuli delivered at 3x C-fibre threshold, 0.5Hz), binned by response latency.

**C** – Raster plot (top) and histogram (bottom) showing responses of the same WDR neuron to mechanical stimulation of the hindpaw with a graded series of vFH. Each stimulation had a duration of 10s, with a 10s inter-stimulus interval. Stimulus presentation (**red**) and withdrawal (**blue**) are illustrated with arrows for the 8g stimulus.

**D** – Raster plot (top) and histogram (bottom) showing reduced responses of the same WDR neuron to the same series of vFH stimuli after cumulative morphine dosing (0.5, 2.5, & 3.5mg.kg^-1^). Data were collected 50mins after the final morphine dose

**Supplemental Figure 5 – Effects of anxiety & OA-like pain on neuronal responses to nociceptive input**


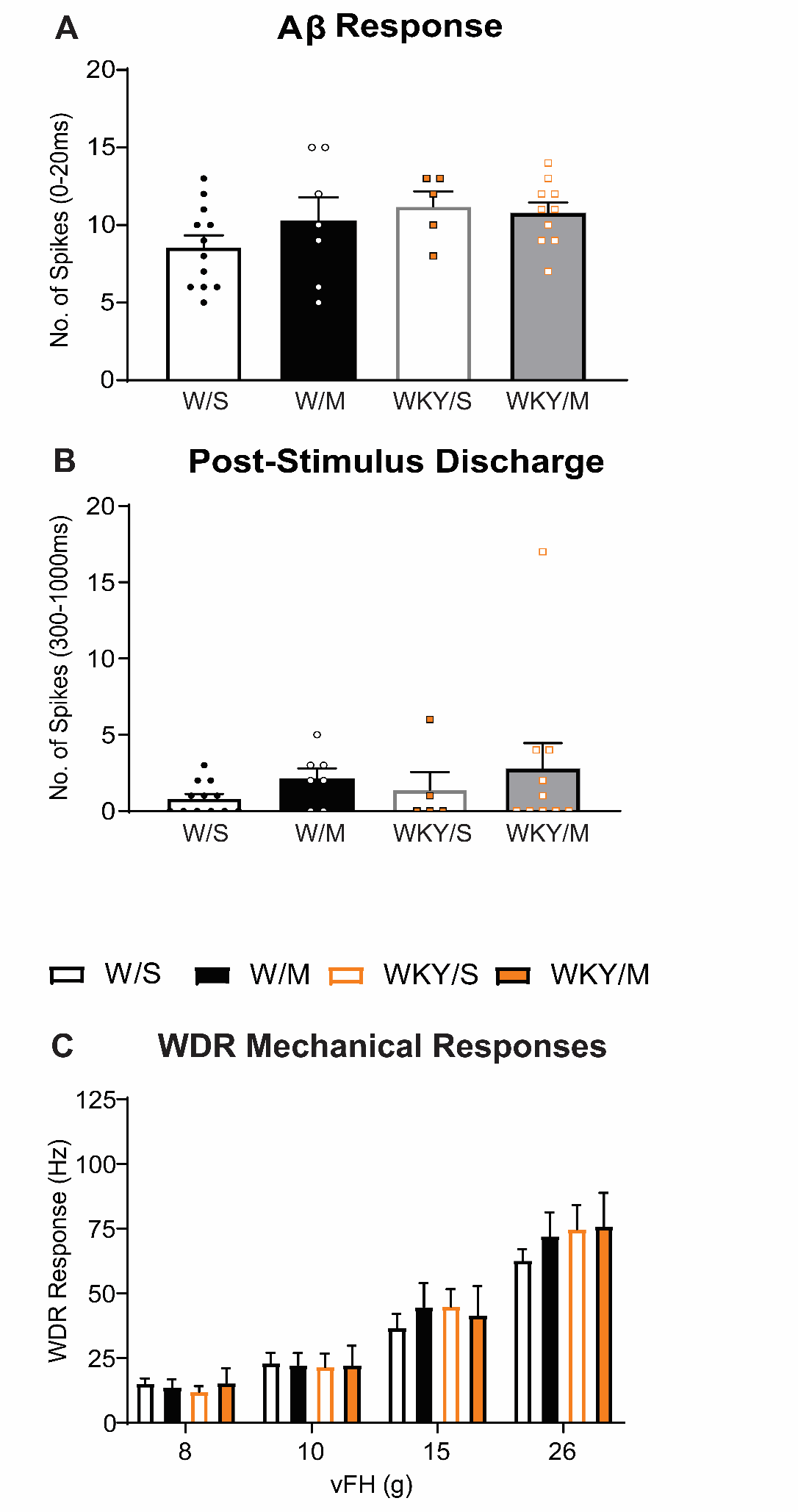


WDR responses to electrical stimulation at 3 x C fibre threshold binned by the Aβ fibre latency (**A**, 0-20ms) and post-discharge (**B**, >300ms). Data represent the average number of action potentials recorded within each post-stimulus time frame, with individual data points shown, and bars representing mean values and error bars the SEM. No significant differences, 2-way ANOVA with Tukey’s *post-hoc* multiple comparison test (**Supplemental Table 6**).

**C:** There were no significant differences in WDR responses to a range of mechanical stimuli applied to the hindpaw receptive field. 2-way ANOVA with Tukey’s *post-hoc* multiple comparison test (**Supplemental Table 6**).

**Supplemental Figure 6 – Expanded Western blot data
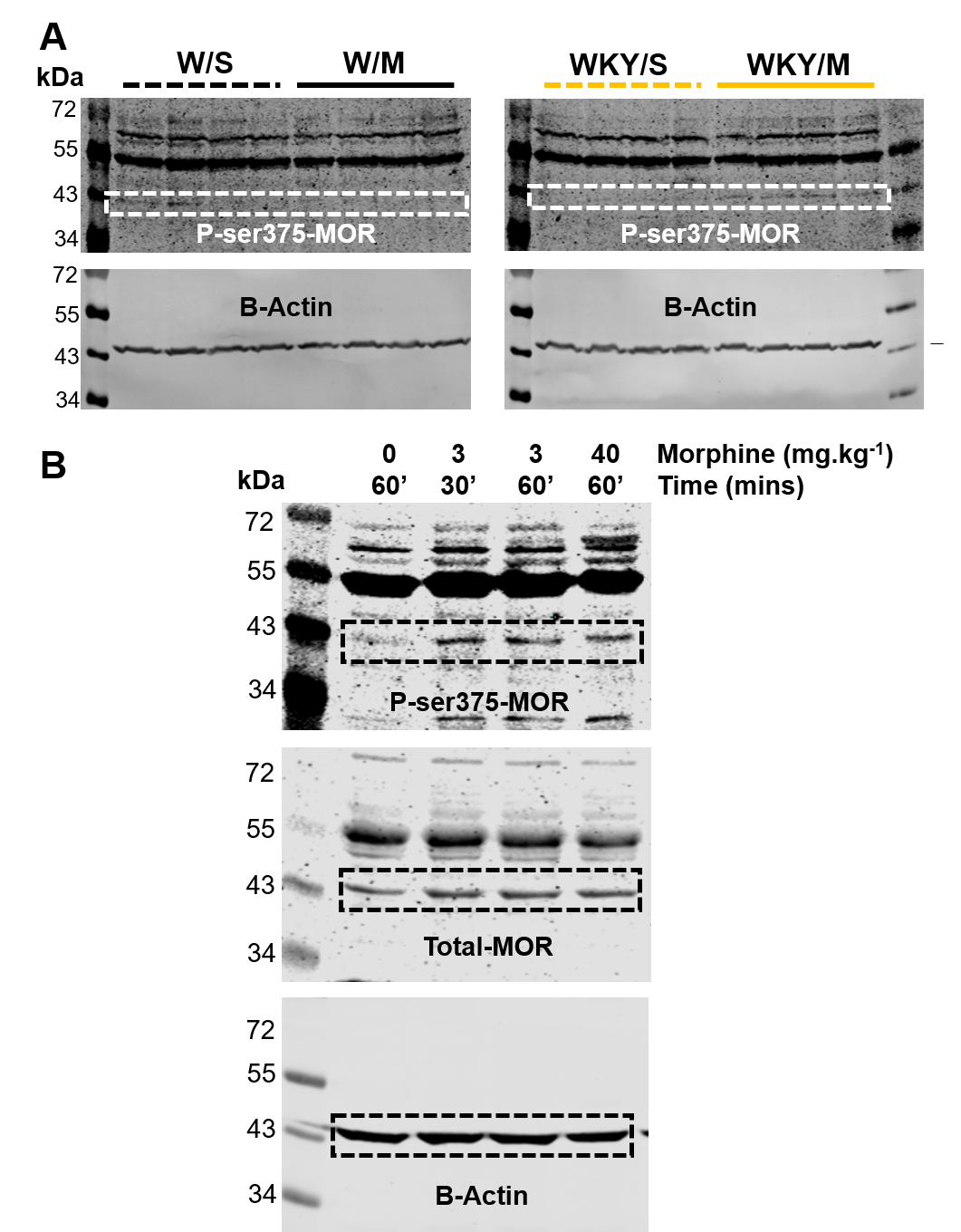
**

**A:** An expanded version of the blots shown in Figure 5A, showing molecular weight markers and position of P-ser-375-MOR bands at the expected molecular weight of ~44kDa, and B-actin loading control at 42kDa.

**B:** - Positive control experiment, comparing effects of acute systemic administration of morphine (3 or 40mg.kg-1, i.p.) or saline in WKY rats on MOR in the dorsal horn of the spinal cord at 30 or 60mins after treatment. P-ser-375-MOR (top) was at the lower limit of detection in SCDH tissue from the saline-treated animal (0mg.kg^-1^ morphine), but markedly increased following morphine treated. Additional blots demonstrate expression of total MOR (middle) and β-actin (bottom), confirming that equal protein concentrations were loaded for each sample. Both the P-ser375-MOR and total MOR antibodies produce multiple higher molecular weight bands which may correspond to multiplexed forms of MOR, or result from non-specific binding. As the identity of these bands is not known, these were not quantified.

**Supplemental References**

[1] Arvidsson U, Riedl M, Chakrabarti S, Lee J, Nakano A, Dado R, Loh H, Law P, Wessendorf M, Elde R. Distribution and targeting of a mu-opioid receptor (MOR1) in brain and spinal cord. J Neurosci 1995;15(5):3328-3341.

[2] Brewer KL, Baran CA, Whitfield BR, Jensen AM, Clemens S. Dopamine D3 receptor dysfunction prevents anti-nociceptive effects of morphine in the spinal cord. Front Neural Circuits 2014;8(62).

[3] Burston JJ, Valdes AM, Woodhams SG, Mapp PI, Stocks J, Watson DJG, Gowler PRW, Xu L, Sagar DR, Fernandes G, Frowd N, Marshall L, Zhang W, Doherty M, Walsh DA, Chapman V. The impact of anxiety on chronic musculoskeletal pain and the role of astrocyte activation. PAIN 2019;160(3):658-669.

[4] Drake CT, Milner TA. Mu opioid receptors are in somatodendritic and axonal compartments of GABAergic neurons in rat hippocampal formation. Brain Res 1999;849(1):203-215.

[5] Laureano DP, Dalle Molle R, Alves MB, Luft C, Desai M, Ross MG, Silveira PP. Intrauterine growth restriction modifies the hedonic response to sweet taste in newborn pups – Role of the accumbal μ-opioid receptors. Neuroscience 2016;322:500-508.
